# Identification of Jmjd3 as an Essential Epigenetic Regulator of *Hox* Gene Temporal Collinear Activation for Body Axial Patterning in Mice

**DOI:** 10.3389/fcell.2021.642931

**Published:** 2021-07-21

**Authors:** Feng Zhang, Xiong Zhao, Runmin Jiang, Yuying Wang, Xinli Wang, Yu Gu, Longyong Xu, Jing Ye, Charlie Degui Chen, Shuangping Guo, Dawei Zhang, Daqing Zhao

**Affiliations:** ^1^State Key Laboratory of Cancer Biology, Department of Pathology, Xijing Hospital, Fourth Military Medical University, Xi’an, China; ^2^Department of Pathology, Air Force Medical Center (Air Force General Hospital), Chinese People’s Liberation Army, Beijing, China; ^3^Department of Orthopedics, Xijing Hospital, Fourth Military Medical University, Xi’an, China; ^4^Department of Thoracic Surgery, Tangdu Hospital, The Fourth Military Medical University, Xi’an, China; ^5^State Key Laboratory of Molecular Biology, Shanghai Key Laboratory of Molecular Andrology, Institute of Biochemistry and Cell Biology, Shanghai Institutes for Biological Sciences, Chinese Academy of Sciences, Shanghai, China; ^6^Department of Otolaryngology, Tangdu Hospital, The Fourth Military Medical University, Xi’an, China

**Keywords:** Jmjd3, Ezh2, H3K27me3, homeotic transformation, temporal collinearity of *Hox* gene activation, chondrogenic cells

## Abstract

Body axial patterning develops *via* a rostral-to-caudal sequence and relies on the temporal colinear activation of *Hox* genes. However, the underlying mechanism of *Hox* gene temporal colinear activation remains largely elusive. Here, with small-molecule inhibitors and conditional gene knockout mice, we identified Jmjd3, a subunit of TrxG, as an essential regulator of temporal colinear activation of *Hox* genes with its H3K27me3 demethylase activity. We demonstrated that Jmjd3 not only initiates but also maintains the temporal collinear expression of *Hox* genes. However, we detected no antagonistic roles between Jmjd3 and Ezh2, a core subunit of PcG repressive complex 2, during the processes of axial skeletal patterning. Our findings provide new insights into the regulation of *Hox* gene temporal collinear activation for body axial patterning in mice.

## Introduction

The development of the mammalian body axis is a continuous process *via* a rostral-to-caudal sequence (Deschamps and Duboule, 2017; Mongera et al., 2019). The morphological characteristics of each part of the body axis are mainly determined by a “Hox code” (Kessel and Gruss, 1991; Alexander et al., 2009; Wellik, 2009). *Hox* genes were first discovered in *Drosophila* (Lewis, 1978). Mammals have 39 Hox genes, which are divided into four clusters. Each cluster locates on different chromosomes and has 9–11 *Hox* genes. According to the sequence homologies and their position within the cluster, the *Hox* genes are divided into 1–13 paralog groups (PGs) (Casaca et al., 2014). The function of *Hox* genes in the same PG is similar and can be mutually replaced (Wellik, 2009). The expression of *Hox* genes in mammals is characterized by spatial collinearity, which means that the sequence of the *Hox* gene cluster from 3′ to 5′ on chromosomes corresponds to the expression domain of *Hox* genes *via* a rostral-to-caudal sequence on the body axis (Deschamps and van Nes, 2005; Montavon and Soshnikova, 2014; Deschamps and Duboule, 2017). Thus, the inactivation of 3′ *Hox* genes leads to the altered identities in the rostral side of the body axis, while the loss of 5′ *Hox* genes causes the morphological change of caudal segments (Deschamps and van Nes, 2005; Montavon and Soshnikova, 2014; Deschamps and Duboule, 2017). Interestingly, establishment of spatial collinear *Hox* gene expression relies on the temporal collinear activation of *Hox* genes in mice (Deschamps and van Nes, 2005; Montavon and Soshnikova, 2014; Deschamps and Duboule, 2017). In other words, *Hox* genes become activated in a time order corresponding to their location within the clusters (Montavon and Soshnikova, 2014; Deschamps and Duboule, 2017; Krumlauf, 2018). At E7.2, the 3′ *Hox* genes are firstly expressed in the posterior primitive streak, and over time, the expression of *Hox* genes gradually transited to the more 5′ *Hox13* PGs at E9.5 (Deschamps and van Nes, 2005; Soshnikova and Duboule, 2009; Montavon and Soshnikova, 2014; Deschamps and Duboule, 2017). Synchronization with the initiation order of *Hox* gene activation and *Hox* gene expression domains start to spread rostrally from the primitive streak, then the expression of 3′ *Hox* genes spreads to the more rostral side, while the 5′ *Hox* genes were expressed in the more caudal segments (Deschamps and van Nes, 2005; Montavon and Soshnikova, 2014; Deschamps and Duboule, 2017). Temporal collinear abnormalities of *Hox* genes will lead to corresponding morphological alterations of the *Hox* gene that spatially expresses the domain *via* a rostral-to-caudal sequence on the body axis. For example, deletion of the *Hoxc8* enhancer causes a transient delay in the initial transcription of the *Hoxc8* gene and results in homeotic transformations, which phenocopies the axial defects of the *Hoxc8*-null mutant in mice (Juan and Ruddle, 2003). On the contrary, a precocious expression of *Hox13* genes causes premature arrest of posterior axial growth (Young et al., 2009). Therefore, the accurately spatiotemporal expression of *Hox* genes is essential for correct body axis patterning.

The initiation of body axis patterning is proposed by signal molecules. For example, embryos *of Wnt3* inactivation do not express any *Hox* genes (Liu et al., 1999). Pre-gastrulation embryos exposed to the Wnt agonist precociously express *Hoxa1* and *Hoxb1* (Neijts et al., 2016). Homozygous *Wnt-3a* mutant mice exhibit homeotic transformations in the vertebrae along their entire body axis (Ikeya and Takada, 2001). These results suggest that Wnt3 is required for the *Hox* gene activation and body axis patterning. After initiation of expression, the transcriptional pattern of *Hox* genes is maintained by the antagonistic complex Polycomb (PcG) and Trithorax (TrxG) (Geisler and Paro, 2015; Piunti and Shilatifard, 2016). PcG generally maintain *Hox* gene repression, while TrxG counteract PcG and maintain the active expression state of *Hox* genes (Mallo and Alonso, 2013; Montavon and Soshnikova, 2014). PcG genes were originally identified in *Drosophila*. Mutations in PcG genes cause ectopic *Hox* gene expression, resulting in posterior homeotic transformations (Simon et al., 1992; Montavon and Soshnikova, 2014). TrxG genes were discovered to suppress the homeotic phenotypes displayed by PcG mutants (Kennison and Tamkun, 1988). Biochemical studies have revealed that PcG and TrxG form multiprotein complexes containing both histone methyltransferase and demethylases activities, respectively. For example, Ezh2 (Kmt6b) is a core subunit of PcG repressive complex 2 (PRC2) and inhibits *Hox* gene transcription by establishment of repressed histone modification H3K27 trimethylation (H3K27me3) with its histone methyltransferase activity (Cao et al., 2002; Czermin et al., 2002; Kuzmichev et al., 2002; Muller et al., 2002). H3K27me3 can be removed by histone demethylases Utx (Kdm6a) and Jmjd3 (Kdm6b) (Agger et al., 2007; De Santa et al., 2007; Hong et al., 2007; Lan et al., 2007), which belong to the TrxG group for the biochemical association with Mll2 or RbBP5 (De Santa et al., 2007; Lee et al., 2007). Interestingly, a ChIP-seq study found that the temporal collinear activation of *Hox* genes was accompanied with a sequential elimination of repressive marker H3K27me3 on the *Hox* genes (Soshnikova and Duboule, 2009; Noordermeer et al., 2014). Using high-resolution chromatin conformation capture methodology, Noordermeer et al. detected newly activated *Hox* genes progressively clustering into a transcriptionally active compartment from a transcriptionally inactive *Hox* gene cluster after transcription starts; thus, the *Hox* gene clusters switch to a bimodal 3D organization. This transition in spatial configurations coincides with the dynamical erase of H3K27me3 on the *Hox* gene clusters (Noordermeer et al., 2011, 2014). However, to date a rare epigenetic regulator for temporal collinear activation of *Hox* genes has been identified. Moreover, whether PcG and TrxG complexes antagonistically adjust the temporary collinear activation of *Hox* genes is not strictly examined in mammals.

Due to obvious morphological differences of each part, axial skeleton is an ideal model to study the relation between *Hox* gene expression and morphogenesis of the body axis (Wellik, 2009). Here, with the axial bone as a model, combined with conditional genetic deletion and biochemical techniques, we identified Jmjd3 as an essential epigenetic regulator of *Hox* gene temporal colinear activation for mouse body axial patterning. Furthermore, we detected no dynamical interplay between Jmjd3 and Ezh2 during the process of axial skeletal patterning in mice.

## Results

### Jmjd3 Is a Potential Candidate Epigenetic Regulator of *Hox* Gene Temporal Collinear Activation

The temporal collinear activation of mouse *Hox* genes occurs at the embryonic stage of E7.2–E9.5 days (Deschamps and van Nes, 2005; Soshnikova and Duboule, 2009; Deschamps and Duboule, 2017). To identify the epigenetic regulators of *Hox* gene temporal collinear activation, we profiled the expression of 17 histone methyltransferases and 28 histone demethylases in E7.5, E8.5, and E9.5 embryonic tissues. Among these tissues, E7.5 tissue was from the whole mouse embryos, while E8.5 and E9.5 tissues were from the trunk of embryos, as previously described (Soshnikova and Duboule, 2009). Compared with E7.5 embryos, real-time reverse transcriptase-quantitative polymerase chain reaction (RT-qPCR) revealed that, except for a few silenced genes, most histone methyltransferases were significantly activated in E9.5 embryos instead of either E7.5 or E8.5 embryonic tissues (Supplementary Figure 1A). Because most *Hox* genes had been activated at E9.5 days (Deschamps and van Nes, 2005; Soshnikova and Duboule, 2009; Deschamps and Duboule, 2017), these results indicated that most histone methyltransferases might not be essential for the temporal collinear activation of *Hox* genes. Interestingly, although half of the 28 histone demethylases genes were silenced, *Kdm6b* (*Jmjd3)* was significantly successively activated during the E7.5–E9.5 stages (Supplementary Figure 1B). Jmjd3 is a potent H3K27me3 demethylase (Agger et al., 2007; De Santa et al., 2007; Lan et al., 2007). A previous report showed that the temporal collinear activation of *Hox* genes was accompanied with the progressive elimination of H3K27me3 on *Hox* genes (Soshnikova and Duboule, 2009). Therefore, these results suggest that Jmjd3 is a potential candidate for the regulation of *Hox* gene temporal collinear activation.

### Jmjd3 Rather Than Utx Is Required for Mouse Body Axis Patterning

Both Jmjd3 and Utx are potent H3K27me3 demethylases. Previously, Utx was demonstrated to be required for the body axis patterning of zebrafish and *Drosophila* (Lan et al., 2007; Copur and Muller, 2013, 2018). To test the roles of Utx and Jmjd3 during body axis patterning in mice, we conditionally delete *Utx* or *Jmjd3* in uncommitted mesenchymal cells of mice, respectively. Skeleton assays revealed, in addition to the fusions of C1 and C2 and sternal asymmetries, that no obvious homeotic transformations in the vertebrae along the entire body axis in *Utx*^*fl*/*Y*^;*Prx1*-*Cre* (0/25) or *Utx*^*fl*/*fl*^;*Prx1*-*Cre* (0/22) mice (Supplementary Figure 2 and Table 1) was detected. This indicated Utx is not required for mouse axial skeletal patterning. However, the phenotypes of *Jmjd3*^fl/fl^;*Prx1*-*Cre* mice were very similar to those of *Jmjd3*^fl/fl^;*EIIa-Cre* mice as previously described for E18.5 (Zhang et al., 2015). Both embryos have dwarfism with thoracic kyphosis (Supplementary Figure 2; Zhang et al., 2015). The complete fusions of C1 and C2 (14/14) were detected in *Jmjd3*^fl/fl^;*Prx1*-*Cre* mice. Homeotic transformations in the vertebrae along the whole-body axis in *Jmjd3*^fl/fl^;*Prx1*-*Cre* mice were visibly detected, including anterior transformation of C2 to C1 (an extra ventral tubercle shared by fused C1 and C2, 14/14), T1 to C7 (T1 without ribs or with incomplete ribs, 7/14), T3 to T2 (longest spinous process on T3 instead of T2,3/14), L1 to T13 (L1 with a pairs of complete or incomplete ribs, 14/14), and S1 to T6 (S1 without bilateral or unilateral iliac bones, 10/14) (Supplementary Figure 2 and Table 1). In addition, some anterior transformations, such as L1 to T13 (7/10), were also detected in *Jmjd3*^*fl/*+^;*Prx1*-Cre embryos, indicating the dose effect of Jmjd3 on axial skeletal patterning (Supplementary Figure 2 and Table 1). Consistently, RNA-seq assays indicated that Jmjd3 loss in uncommitted mesenchymal cells mildly decreased the expression of *Hox3*–*5* PGs but markedly reduced the mRNA level of *Hox6*–*10* PGs compared to WT in E8.5 littermates (Supplementary Figures 3, 4 and Supplementary Table 2). Therefore, these results indicated that Jmjd3 rather than Utx is required for mouse body axis patterning.

**TABLE 1:** ^*a*^Homeotic transformations of axial skeletons with various genotypes.

Genotypes	Time points	Total (*n*)	Homeotic transformations of axial skeletons						
			^b^C2 to C1	^c^T1 to C7	^d^T3 to T2	^e^T8 to T7	^f^L1 to T13	^g^S1 to L6	^h^L6 to S1
^i^Control		60							4 (6.7%)
*Jmjd3^*fl/*+^;Prx1-Cre*		10	1 (10.0%)				7 (70.0%)		
*Jmjd3^*fl/fl*^;Prx1-Cre*		14	14 (100%)	7 (50.0%)	3 (21.4%)		14 (100%)	10 (100%)	
*Utx^*fl/Y*^;Prx1-Cre*		25							1 (4.0%)
*Utx^*fl/fl*^;Prx1-Cre*		22							2 (9.0%)
*Ezh2^*fl/*+^;Prx1-Cre*		17							10 (58.8%)
*Ezh2^*fl/fl*^;Prx1-Cre*		15							7 (46.7%)

*Jmjd3^*fl/fl*^;Col2a1-Cre^*ERT2*^*	E8.5	18	7 (38.9%)	10 (55.6%)	6 (33.3%)	16 (88.9%)	18 (100%)	9 (50%)	
	E9.5	17				15 (88.2%)	17 (94.4%)	13 (76.5%)	
	E10.5	21				3 (14.2%)	4 (19.0%)		

*Ezh2^*fl/fl*^;Col2a1-Cre^*ERT2*^*	E8.5	20							12 (60.0%)
	E9.5	21							10 (47.6%)
	E10.5	16							7 (43.8%)

*Jmjd3^*fl/fl*^;Ezh2^*fl/fl*^;Col2a1-Cre^*ERT2*^*	E8.5	12	5 (41.7%)	8 (66.7%)	5 (41.7%)	10 (83.3%)	12 (100%)	9 (75.0%)	
	E9.5	10				8 (80.0%)	9 (90.0%)	5 (50.0%)	
	E10.5	25				4 (16.0%)	5 (20.0%)	3 (12.0%)	

*^*a*^Numbers of mice in each category are shown. Both unilateral and bilateral abnormalities were included in axial skeleton analysis. Number of sacral vertebrae was not counted because the cartilaginous linkages between the sacral vertebrae S3 and S4 are often only very fine at birth.*

*^*b*^An extra ventral tubercle shared by fused C1 and C2.*

*^*c*^T1 without ribs or with incomplete ribs.*

*^*d*^Longest spinous process on T3 instead of T2.*

*^*e*^T8 with unilateral or bilateral vertebrosternal ribs.*

*^*f*^L1 with a pairs of complete or incomplete ribs.*

*^*g*^S1 without bilateral or unilateral iliac bones.*

*^*h*^L6 with bilateral or unilateral iliac bones.*

*^*i*^Mice of control group including the genotypes of *Col2a1-Cre^*ERT2*^, Jmjd3^*fl/fl*^, Utx^*fl/fl*^*, and *Ezh2^*fl/fl*^, each* genotype contain 15 mice.*

*C, cervical; T, thoracic; L, lumbar; S, sacral; WT, wild type.*

### Jmjd3 Temporally Regulates the Axial Skeletal Patterning of Mice With Its H3K27me3 Demethylase Activity

**FIGURE 1:**
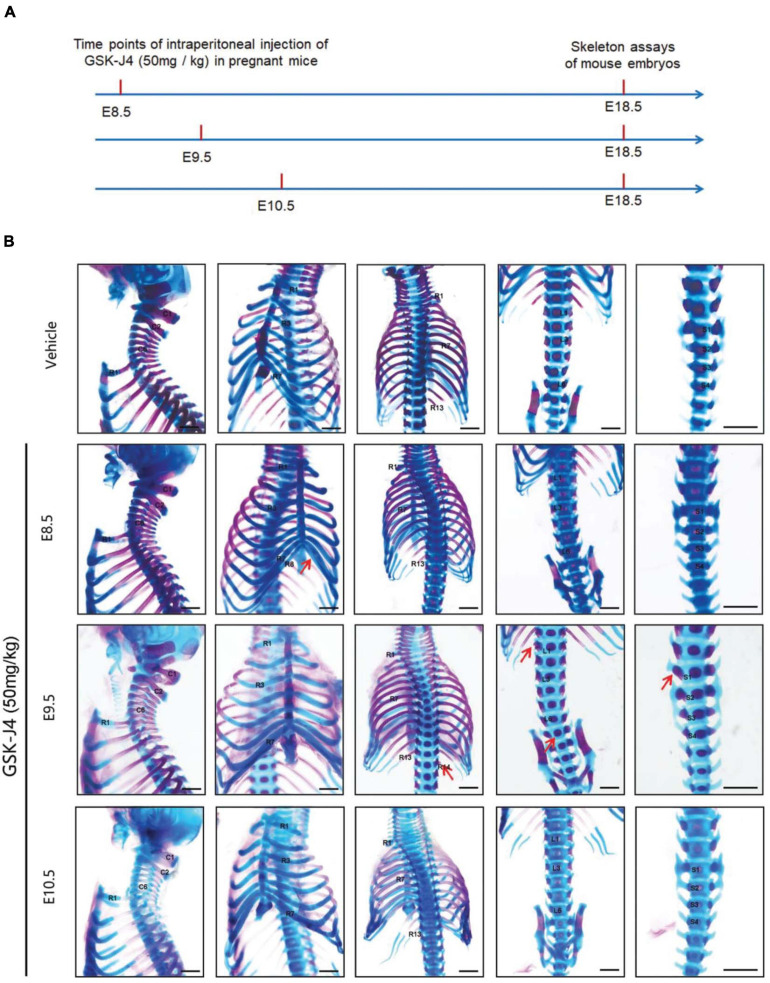
Homeotic transformations by GSK-J4 treatment at the time points of E8.5, E9.5, or E10.5. **(A)** Experimental design for studying the roles of GSK-J4 on body axis patterning. **(B)** Embryos had been exposed to GSK-J4 at the time points of E8.5, E9.5, or E10.5 as indicated. Skeletal phenotypes were prepared by removing forelimbs and hindlimbs. The red arrow indicates homeotic transformations of axial skeleton induced by GSK-J4. Frequencies of homeotic transformations are given in Table 1. The cervical (C), rib (R), lumbar (L), and sacral (S) vertebrae are numbered. For schematic explanation, see Supplementary Figure 9. Scale bar, 1 mm.

*Jmjd3* deletion in uncommitted mesenchymal cells leads to the homeotic transformation in the vertebrae along the whole-body axis (Supplementary Figure 2 and Table 1). To test whether Jmjd3 temporally regulates the axial skeletal patterning with its H3K27me3 demethylase activity, we tested the role of GSK-J4, an inhibitor of Jmjd3 (Ntziachristos et al., 2014), on body axis patterning *in vivo*. One dose of GSK-J4 (50 mg/kg) treatment at E8.5 induced only anterior transformation of T8 to T7 (26/26) at the middle part of the trunk but not at the cervical, lumbar, and sacral segments of E18.5 mice (Figures 1A,B and Table 2). GSK-J4 treatment at E9.5 produced homeotic transformations of L1 to T13 (15/15) and S1 to T6 (14/15) in the caudal half of the vertebral column of E18.5 mice (Figures 1A,B and Table 2). GSK-J4 treatment at E10.5 produced no obvious homeotic transformations in E18.5 mouse embryos (Figures 1A,B and Table 2). These were consistent with previous reports that *Hox* genes of the middle part or 5′ end of the *Hox* gene cluster were activated at E8.5 or E9.5, respectively (Deschamps et al., 1999; Kmita and Duboule, 2003; Deschamps and van Nes, 2005; Soshnikova and Duboule, 2009). GSK-J4 treatment at E8.5 or E9.5 induced anterior transformation at the corresponding part of axial skeletal patterning, strongly supporting that Jmjd3 temporally regulates the axial patterning of mice with its H3K27me3 demethylase activity.

**TABLE 2:** Skeletal analysis of E18.5 mouse embryos after treatment by GSK-J4 (50 mg/kg) or GSK-126 (100 mg/kg) at the indicated time points^a^.

Inhibitors	Vehicle	GSK-J4				GSK-126	

Time points		E8.5	E9.5	E10.5	E8.5	E9.5	E10.5

Total number of mice	46	26	15	25	29	17	16
**Homeotic transformation of axial skeletons**					l		
^b^T8 to T7		26 (100%)					
^c^L1 to T13			15 (100%)				
^d^S1 to L6			14 (93.3%)				
^e^L6 to S1	2 (4.3%)	1 (3.8%)	0 (0%)	1 (4%)	9 (31.0%)	5 (29.4%)	4 (25.0%)

*^*a*^Numbers of mice in each category are shown, and all mice were of hybrid background. Both unilateral and bilateral abnormalities were included in axial skeleton analysis. Number of sacral vertebrae was not counted because the cartilaginous linkages between the sacral vertebrae S3 and S4 are often only very fine at birth.*

*^*b*^T8 with unilateral or bilateral vertebrosternal ribs.*

*^*c*^L1 with a pairs of complete or incomplete ribs.*

*^*d*^S1 without bilateral or unilateral ilial bones.*

*^*e*^L6 with bilateral or unilateral ilial bones.*

*C, cervical; T, thoracic; L, lumbar; S, sacral.*

### Jmjd3 Regulated *Hox* Gene Temporal Collinear Activation With Its H3K27me3 Demethylase Activity

**FIGURE 2:**
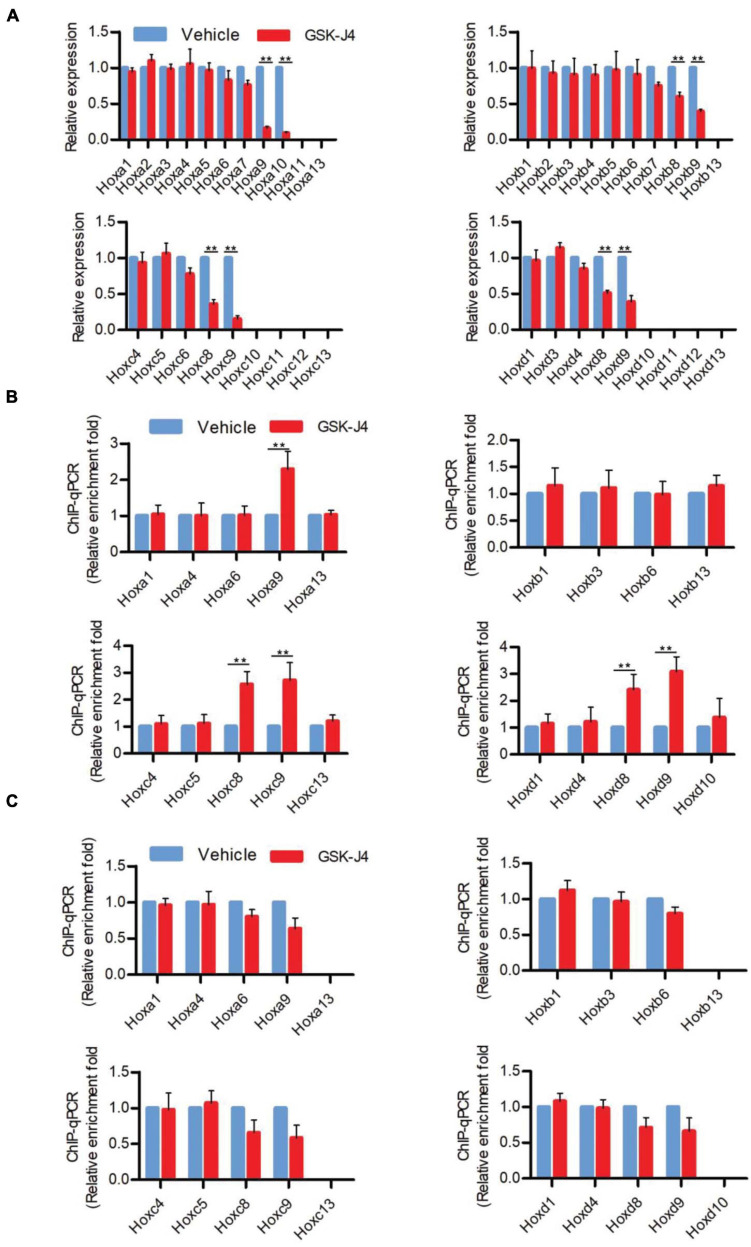
GSK-J4 inhibits transcription of *Hox* genes by blocking the H3K27me3 demethylase activity of Jmjd3 in E8.5 mouse embryos. **(A)** The mRNA levels of *Hox* genes were determined by RT-qPCR in E8.5 embryos, which were treated by vehicle or GSK-J4 (50 mg/kg) 12 h before. **(B,C)** The ChIP-qPCR assays with antibodies specific for H3K27me3 **(B)** or Jmjd3 **(C)** on the *Hox* genes in E8.5 embryos, which were exposed to vehicle or GSK-J4 (50 mg/kg) 12 h before. Bars indicate triplicate PCR reactions ± SD. Representative results of three independent experiments are shown. *p < 0.05, **p < 0.01.

In murine embryos, *Hox* genes at the 3′ end of *Hox* clusters are first activated at E7.2 (Deschamps and Wijgerde, 1993; Forlani et al., 2003) and the last *Hox* genes at the 5′ end of *Hox* clusters complete the transcription at around E9.5 (Deschamps et al., 1999; Kmita and Duboule, 2003; Deschamps and van Nes, 2005; Soshnikova and Duboule, 2009). Subsequently, the transcriptional states of *Hox* genes at each segment are memorized along the rostral-to-caudal axis and ultimately determine body axial patterning (Kmita and Duboule, 2003; Deschamps and van Nes, 2005). A previous investigation indicated that the temporal collinear activation of *Hox* genes was accompanied with a sequential elimination of H3K27me3 on the *Hox* genes during the process of body axis patterning (Soshnikova and Duboule, 2009; Noordermeer et al., 2014). To test whether Jmjd3 regulates the *Hox* gene temporal collinear activation with its H3K27me3 demethylase activity, we firstly examined the mRNA level of *Hox* genes after GSK-J4 treatment at different time points. Consistent with the anterior transformation of T8 to T7 in the middle part instead of the cervical, lumbar, and sacral segments of mouse embryos induced by GSK-J4 at E8.5 (Figures 1A,B and Table 2), GSK-J4 treatment at E8.0 significantly reduced the mRNA level of *Hox*8–9 PGs, which are responsible for the establishment of the T7 and T8 patterns (Favier and Dolle, 1997; Wellik, 2009), but did not affect the mRNA levels of other *Hox* PGs, such as *Hox*1–6 PGs in E8.5 mouse embryos (Figure 2A and Supplementary Figure 5A). Correspondingly, GSK-J4 treatment at E9.0 significantly reduced the mRNA level of *Hox11*–*13* PGs (Supplementary Figures 5A, 6A) in E9.5 mouse embryos, which was consistent with the GSK-J4 treatment at E9.5 which produced anterior transformations of the vertebrae only at the caudal part of the vertebral column, including L1 to T13 and S1 to T6 (Figures 1A,B and Table 2). To investigate the reason for the mRNA level reduction on *Hox*8–9 PGs in E8.5 embryos or on *Hox11*–*13* PGs in E9.5 embryos by GSK-J4 treatment, we examined the H3K27me3 and Jmjd3 levels on the *Hox* genes by ChIP-qPCR assays. GSK-J4 treatment at E8.0 or E9.0 significantly increased the level of H3K27me3 on the gene body of *Hox*8–9 PGs in E8.5 embryos (Figure 2B and Supplementary Figures 5A, 13) or *Hox*10–13 PGs in E9.5 embryos, respectively (Supplementary Figures 5A, 6B, 13). Therefore, the mRNA level reduction on *Hox*8–9 PGs in E8.5 embryos or on *Hox11*–*13* PGs in E9.5 embryos by GSK-J4 treatment was associated with the increased level of H3K27me3 on corresponding *Hox* genes. That is, Jmjd3 regulated *Hox* gene temporal collinear activation with its H3K27me3 demethylase activity. In addition, at E8.5 embryos, the signal of Jmjd3 was detected on *Hox*1–9 PGs but not *Hox*10–13 PGs by ChIP-qPCR assays (Figure 2C and Supplementary Figure 13). At E9.5, the signal of Jmjd3 was detected on the whole *Hox*1–13 PGs (Supplementary Figures 6C, 13). These results indicated that Jmjd3 bound to *Hox* genes with a temporal collinear manner and was consistent with *Hox* gene temporal collinear activation.

### Jmjd3 Temporally Regulated Axial Skeletal Patterning in Chondrogenic Cells

**FIGURE 3:**
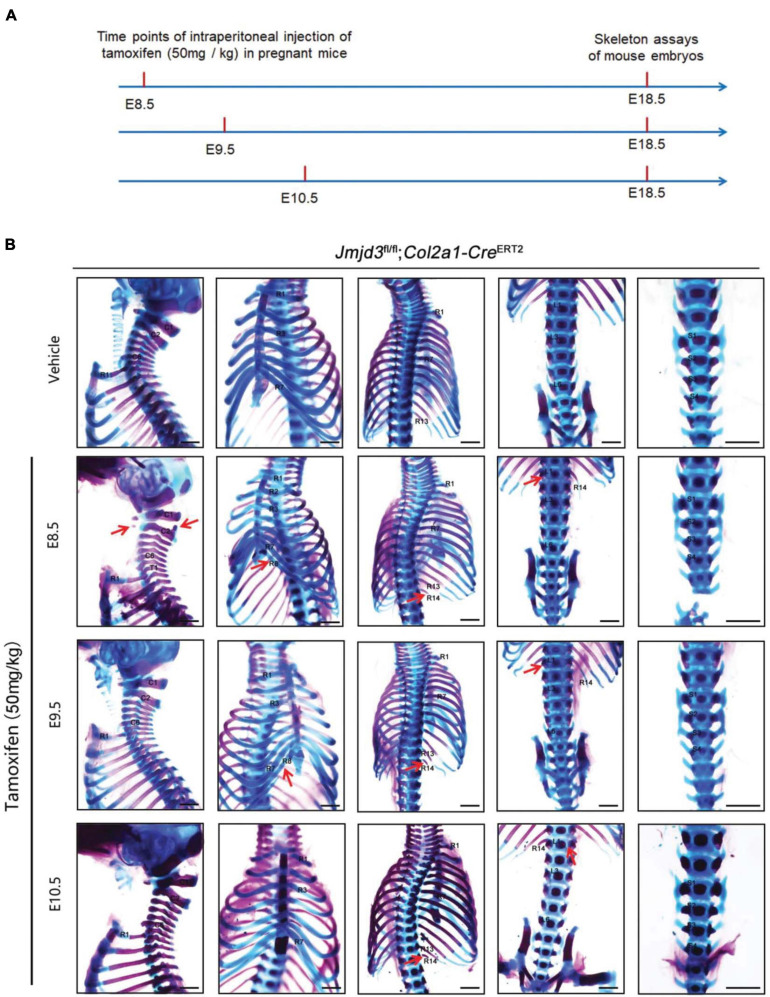
Homeotic transformations by tamoxifen induced *Jmjd3* conditional knockout in *Col2a1* expressing chondrogenic cells at E8.5, E9.5, and E10.5 days. **(A)** Experimental design for studying the roles of Jmjd3 in chondrogenic cells on body axis patterning. **(B)** Skeletal phenotypes were prepared from E18.5 mouse embryos. The red arrow indicates homeotic transformations of axial skeletons. Frequencies of homeotic transformations are given in Table 1. The cervical (C), rib (R), lumbar (L), and sacral (S) vertebrae are numbered. For schematic explanation, see Supplementary Figure 9. Scale bar, 1 mm.

Axial skeletal formation starts from the differentiation of condensed mesenchymal cells to cartilaginous templates (Karsenty, 2008; Long and Ornitz, 2013). To test whether chondrogenic cells are essential for Jmjd3-mediating axial skeletal patterning in mice, *Jmjd3^*fl/fl*^;Col2a1-Cre^*ERT2*^* mice were generated by crossing mice with *Jmjd3*^*fl/fl*^ and *Col2a1-Cre^*ERT2*^* genotypes. The expressing Cre recombinase in chondrogenic cells of *Col2a1-Cre^*ERT2*^* mice can be activated by tamoxifen. *Jmjd3*^*fl/fl*^;*Col2a1-Cre^*ERT2*^* pregnant mice were intraperitoneally injected by tamoxifen in a single dose (50 mg/kg) at a time point from E8.5 to E10.5. Interestingly, different from the phenotypes of GSK-J4 treatment at E8.5, *Jmjd3* knockout in chondrogenic cells at E8.5 induced more broadness and frequency of axial skeletal abnormalities along the whole-body axis of E18.5 embryos, primarily exhibiting anterior transformation of C2 to C1 (7/18), T1 to C7 (10/18), T3 to T2 (6/18), T8 to T7 (16/18), L1 to T13 (18/18), and S1 to T6 (9/18) (Figures 3A,B and Tables 1, 2). Similarly, different from the phenotypes induced by GSK-J4 treatment at E9.5, *Jmjd3* knockout by tamoxifen at E9.5 induced homeotic transformations of T8 to T7 (15/17), L1 to T13 (17/17), and S1 to T6 (13/17) at the thoracic, lumbar, and sacral regions of E18.5 mouse embryos (Figures 3A,B and Tables 1, 2). Lastly, although GSK-J4 treatment at E10.5 induced no visible homeotic transformations along the axial skeleton (Figures 1A,B and Table 2), *Jmjd3* conditional deletion by tamoxifen at E10.5 produced, though with less frequency, homeotic transformations of T8 to T7 (3/21) and L1 to T13 (4/21) at the thoracic and lumbar segments of E18.5 embryos (Figures 3A,B and Table 1). These results indicate that *Jmjd3* knockout in chondrogenic cells produces more broadness and frequency of homeotic transformations along the whole-body axis than those induced by GSK-J4 at the same time points.

### Jmjd3 Temporally Activated and Maintained *Hox* Gene Expression in Mice

**FIGURE 4:**
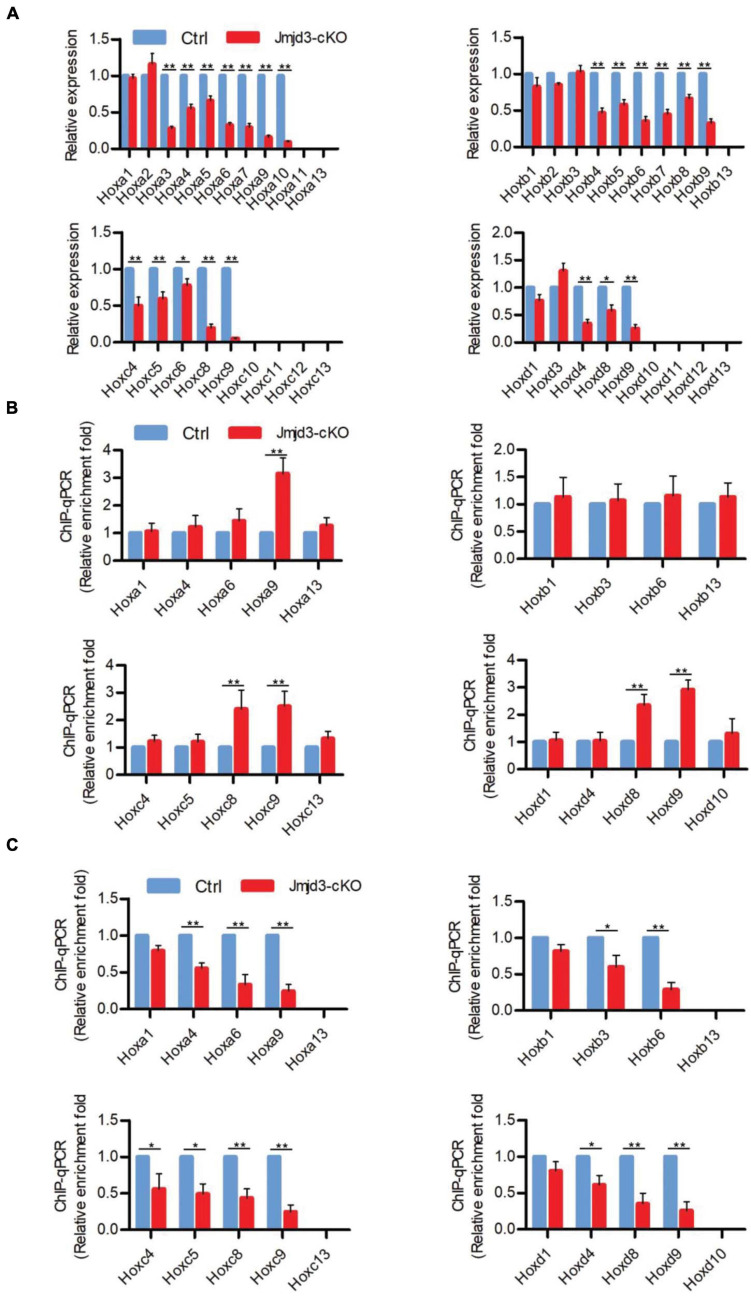
Jmjd3 temporally activated and maintained *Hox* gene expression in mice. **(A)** The mRNA levels of *Hox* genes were determined by RT-qPCR in E8.5 control or *Jmjd3*-cKO embryos, which were treated by tamoxifen (50 mg/kg) 12 h before. **(B,C)** ChIP-qPCR assays with antibodies specific for H3K27me3 **(B)** or Jmjd3 **(C)** on the *Hox* genes in E8.5 control or *Jmjd3*-cKO embryos treated by tamoxifen (50 mg/kg) 12 h before. Bars indicate triplicate PCR reactions ± SD. Representative results of three independent experiments are shown. *p < 0.05, **p < 0.01.

Different from the effects of GSK-J4 treatment, conditional deletion of *Jmjd3* at the same time points in chondrogenic cells produced more broadness and frequency of anterior transformation of axial skeleton (Figures 1A,B, 3A,B and Tables 1, 2). Previous results showed that, additionally as an H3K27m3 demethylase, Jmjd3 maintains a gene expression independent of H3K27m3 demethylase activity in differentiated cells (Miller et al., 2010) or during the process of early embryonic development (Shpargel et al., 2014). Therefore, we speculated that Jmjd3 in chondrogenic cells might not only mediate the *Hox* gene temporal colinear activation but also maintain *Hox* gene expression after their transcription activation. To test the hypothesis, we examined the effect of *Jmjd3* deletion on the mRNA level of *Hox* PGs during body axis patterning. Interestingly, *Jmjd3* knockout by tamoxifen at E8.0 reduced the mRNA level of *Hox3*–*9* PGs in E8.5 embryos (Figure 4A and Supplementary Figure 5B). However, GSK-J4 treatment at E8.0 only decreased the mRNA level of *Hox8*–*9* PGs in E8.5 embryos (Figure 2A and Supplementary Figure 5A). Knockout of *Jmjd3* at E9.0 by tamoxifen significantly decreased the expression level of *Hox8*–*13* PGs (Supplementary Figures 5B, 7A) in E9.5 embryos, while GSK-J4 treatment at E9.0 only reduced the transcriptional level of *Hox11*–*13* PGs in E9.5 embryos (Supplementary Figures 5A, 6A). *Jmjd3* deletion in chondrogenic cells obviously affected more *Hox* PG expression than those by GSK-J4 treatment at the same time points. These results were consistent with the fact that *Jmjd3* deletion by tamoxifen produced more broadness and frequency of anterior transformation of axial skeleton than those by GSK-J4 treatment at the same time points (Figures 1A,B, 3A,B). Consistently, tamoxifen instead of GSK-J4 treatment at E8.0 significantly reduced the level of Jmjd3 protein on the *Hox3*–*9* PGs in E8.5 embryos (Figures 2C, 4C and Supplementary Figures 5A,B, 13). However, *Jmjd3* deletion only increased the H3K27me3 level on *Hox8*–*9* PGs instead of *Hox3*–*7* PGs (Figure 4B and Supplementary Figures 5B, 13). Because GSK-J4 treatment also only increased the H3K27me3 level on *Hox8*–*9* PGs but not on *Hox3*–*7* PGs (Figure 2B and Supplementary Figures 5A, 13), these results support that Jmjd3 activates *Hox8*–*9* PGs dependent on its H3K27m3 demethylase activity, but maintains the expression of *Hox3*–*7* PGs independent on H3K27m3 demethylase activity. In the same way, tamoxifen rather than GSK-J4 treatment at E9.0 significantly reduced the Jmjd3 protein level on the *Hox8*–*13* PGs (Supplementary Figures 5B,C, 7C, 13) but only increased the H3K27me3 level on *Hox10*–*13* PGs instead of on *Hox8*–*10* PGs in E9.5 embryos (Supplementary Figures 5B, 6B, 7B, 13). Coherently, GSK-J4 treatment at E9.0 only increased the H3K27me3 level on *Hox10*–*13* PGs but not on *Hox8*–*10* PGs (Supplementary Figures 5A, 6B, 13). These results support that Jmjd3 activates *Hox10*–*13* PGs dependent on H3K27m3 demethylase activity but maintains the expression of *Hox8*–*10* PGs independent on H3K27m3 demethylase activity at E9.5 days. Therefore, we conclude that Jmjd3 temporally activates and maintains *Hox* gene expression in mice.

### Jmjd3 and Ezh2 Do Not Antagonistically Control the Axial Skeletal Patterning in Mice

Previous studies indicated that sequential activation of the *Hox* gene relies on dynamic degradation of transcription repression hallmark H3K27me3 (Soshnikova and Duboule, 2009), which is established by histone methyltransferase Ezh2 (Cao et al., 2002; Muller et al., 2002) and erased by Kdm6 (Utx and Jmjd3) (De Santa et al., 2007; Lan et al., 2007; Lee et al., 2007). However, it is not clear whether an antagonistic interplay between Ezh2 and Jmjd3 occurs in a dynamic fashion during axial skeletal patterning. To explore this question, we crossed *Jmjd3*^*fl/fl*^;*Col2a1*-*Cre*^*ERT2*^ mice and *Ezh2*^*fl/fl*^ mice to generate *Jmjd3*^*fl/fl*^;*Ezh2*^*fl/fl*^;*Col2a1*-*Cre*^*ERT2*^ mice, in which both *Jmjd3* and *Ezh2* could be concurrently deleted in chondrogenic cells after tamoxifen treatment. One dose of tamoxifen treatment (50 mg/kg) at E8.5 induced homeotic transformations including anterior transformation of C2 to C1 (5/12), T1 to C7 (8/12), T3 to T2 (5/12), T8 to T7 (10/12), L1 to T13 (12/12), and S1 to T6 (9/12) at the cervical, thoracic, lumbar, and sacral regions (Supplementary Figures 8A,B, 9A and Table 1) in E18.5 *Jmjd3*^*fl/fl*^;*Ezh2*^*fl/fl*^;*Col2a1*-*Cre*^*ERT2*^ mouse embryos, respectively. However, these abnormalities of axial skeletal patterning were very similar to the phenotypes of *Jmjd3*-deleted mice which were treated by tamoxifen at E8.5 (Figures 3A,B, Supplementary Figures 8A,B, 9A, and Table 1). Consistently, double-knockout *Jmjd3* and *Ezh2* by tamoxifen at E9.5 or E10.5 in chondrogenic cells produced similar patterning abnormalities of axial skeletons to those of *Jmjd3*-deleted mice by tamoxifen at E9.5 or E10.5, respectively (Figures 3A,B, Supplementary Figures 8A,B, 9A, and Table 1). Therefore, these indicated that Jmjd3 and Ezh2 do not antagonistically and dynamically control the axial skeletal patterning of mice. Consistently, GSK-126—an inhibitor of Ezh2—treatment (100 mg/kg) at E8.5, E9.5, or E10.5 produced no broad homeotic transformations in the vertebrae along the entire body axis, except for mild increasing frequency of posterior transformation of L6 to S1 at the sacral region (Supplementary Figure 10 and Table 2). Similarly, posterior transformation of L6 to S1 was the only detected homeotic transformation in *Ezh2*^*fl/fl*^;*Prx1*-Cre (7/15) and *Ezh2^*fl/*+^*;*Prx1*-Cre mice (10/17) (Supplementary Figure 11 and Table 1). Furthermore, L6 to S1 was the only detected posterior homeotic transformation in E18.5 *Ezh2^*fl/fl*^;Col2a1-Cre^*ERT2*^* embryos after tamoxifen treatment at E8.5, E9.5, or E10.5, respectively (Supplementary Figures 12A,B and Table 1). Consistently, *Ezh2* deletion cannot rescue the mRNA level of *Hox6*–*10* PGs by *Jmjd3* knockout (Supplementary Figures 14A,B). Therefore, these results strongly suggested that Jmjd3 and Ezh2 do not antagonistically control the axial skeletal patterning in mice.

## Discussion

In this study, we demonstrated that (1) Jmjd3 regulates axial skeletal patterning and *Hox* gene temporal collinear activation with its H3K27me3 demethylase activity. (2) There is no continuously antagonistic interplay between Jmjd3 and Ezh2 in the control of body axis patterning. The results revealed that Jmjd3 rather than Ezh2 is an essential epigenetic regulator of *Hox* gene temporal collinear activation.

### Jmjd3 Is an Essential Epigenetic Regulator of *Hox* Gene Temporal Collinear Activation

*Hox* gene temporal collinear activation is essential for the development of the body axis (Deschamps and Duboule, 2017). During the past decades, important progress has been made in the molecular regulatory mechanism of *Hox* temporal collinearity activation. For example, genetic knockout experiments confirmed that there are different *cis* regulators at the 3′ and 5′ ends to regulate the temporal collinear activation of *Hox* genes (Tschopp and Duboule, 2011). Moreover, it was found that the 3′ activated *Hox* and the 5′ silenced *Hox* were in different chromatin domains (Noordermeer et al., 2011, 2014). However, it has long been speculated that signal molecules are involved in the temporal collinear activation of *Hox* genes, such as Wnt3a (Ikeya and Takada, 2001), Fgfr1 (Partanen et al., 1998), and Gdf11 (McPherron et al., 1999). However, because these reports were not based on conditional real-time gene knockout experiments, whether these signal molecules are really required for *Hox* gene temporal collinear activation remains in need of further research. For the same reason, Naruse et al. (2017) showed that knockout of *Jmjd3* in germ cells can cause homeotic transformations in mice. However, their methods cannot prove that Jmjd3 is the regulator of *Hox* gene temporal collinear activation. In addition, it has been reported that retinoids regulate *Hox* gene temporal collinear activation *in vitro* (Chambeyron and Bickmore, 2004). However, *in vivo* experiments show that retinoids promote *Hox* gene activation at E7.5 but inhibit *Hox* gene expression at E8.5 (Kessel and Gruss, 1991). Thus, it is impossible for retinoids to regulate the whole process of *Hox* gene temporal collinear activation. In the present experiment, we demonstrated that the H3K27me3 demethylase Jmjd3 is the first identified epigenetic molecule regulating the temporal collinear activation of *Hox* genes. The evidence is as follows: among 28 histone demethylases and 17 histone methyltransferases, only Jmjd3 is successively activated and overexpressed in the development of the body axis during the period of *Hox* gene temporal activation (Supplementary Figures 1A,B). GSK-J4 treatment at different time points induced an alteration of the corresponding parts of the axial bone (Figures 1A,B, Supplementary Figure 9A, and Table 1). Finally, Jmjd3 could initiate *Hox* gene temporal collinear activation by erasing H3K27me3 (Figures 2B, 4B and Supplementary Figure 9B).

### Jmjd3 Not Only Initiates but Also Maintains the Temporal Collinear Expression of *Hox* Genes

Although Jmjd3 and Utx have proved to be H3K27m3 demethylases (Agger et al., 2007; De Santa et al., 2007; Lan et al., 2007; Lee et al., 2007), in some cases, their function did not rely on H3K27m3 demethylase activity. For example, Jmjd3 and Utx play demethylase-independent roles in chromatin remodeling in differentiated cells where the epigenetic profile is already established (Miller et al., 2010). Shpargel et al. (2014) reported that early embryonic H3K27me3 repression can be alleviated in the absence of active demethylation of Jmjd3 and Utx. Here, we found that the activation rather than maintenance of *Hox* gene expression depended on the H3K27m3 demethylase activity of Jmjd3. For instance, we found that the phenotypes induced by GSK-J4 treatment or conditional deletion of Jmjd3 by tamoxifen were very different (Figures 1A,B, 3A,B, Supplementary Figure 9A, and Tables 1, 2). At E8.5, GSK-J4 induced only anterior transformation of T8 to T7 (6/28) in the middle part of the trunk of mice (Figures 1A,B and Table 2). However, *Jmjd3* deletion at E8.5 by tamoxifen induced more extensive homeotic transformations across the whole-body axis, including C2 to C1, T1 to C7, T3 to T2, T8 to T7, L1 to T13, and S1 to T6 (Figures 3A,B and Table 1). RT-qPCR revealed that GSK-J4 treatment at E8.0 only reduced the mRNA level on *Hox8*–*9* PGs (Figure 2A and Supplementary Figure 5A), while *Jmjd3* deletion by tamoxifen at E8.0 decreased the mRNA level of *Hox3*–*10* PGs in E8.5 mouse embryos (Figure 4A and Supplementary Figure 5B). Increasing the level of H3K27me3 on *Hox8*–*9* PGs in mouse embryos by both GSK-J4 treatment and *Jmjd3* deletion indicated that the removal of H3K27me3 on *Hox8*–*9* PGs depends on the Jmjd3 demethylase activity at the time point of E8.5 (Figures 2B, 4B and Supplementary Figures 5A,B). However, the *Jmjd3* deletion instead of GSK-J4 treatment can reduce the mRNA level of *Hox 3*–*5* PGs, on which no alteration of the H3K27me3 level was detected at this time point (Figures 2A,B, 4A,B and Supplementary Figures 5A,B), indicating that the maintaining expression of these *Hox* genes did not require the H3K27me3 demethylase of Jmjd3. In the same way, at E9.5, GSK-J4 only decreased the mRNA level of *Hox11*–*13* PGs and the corresponding caudal homeotic transformations (Figures 1A,B, 2A and Supplementary Figure 5A). However, *Jmjd3* knockout can reduce the mRNA level of not only *Hox11*–*13* PGs but also *Hox8*–*10* PGs (Figure 4A and Supplementary Figure 5B), thus inducing homeotic transformations of the thoracic, lumbar, and sacral regions (Figures 3A,B and Table 2). Accordingly, these results showed that the removal of H3K27me3 on *Hox11*–*13* PGs instead of on *Hox8*–*10* PGs depends on the H3K27me3 demethylase activity of Jmjd3 (Figures 2B, 4B and Supplementary Figure 5B). Therefore, the maintaining expression of *Hox8*–*10* PGs requires Jmjd3 protein rather than its enzyme activity.

### No Continuous Dynamic Interplay Between Jmjd3 and Ezh2 in the Control of Body Axis Patterning

PcG or TrxG group proteins played critical roles in maintaining the expression pattern of *Hox* genes (Mallo and Alonso, 2013; Geisler and Paro, 2015). Mutations in PcG genes lead to ectopic *Hox* gene expression and consequent posterior homeotic transformations, while deletions of TrxG genes result in delayed *Hox* gene expression and consequent anterior homeotic transformations in both *Drosophila* and vertebrates (Mallo and Alonso, 2013; Geisler and Paro, 2015). Therefore, *Hox* gene expression was proposed to be dynamically and antagonistically regulated by PcG and TrxG (Hanson et al., 1999; Klymenko and Muller, 2004; Sheikh et al., 2015). Here, we detected that the homolog of TrxG group Jmjd3 loss results in anterior transformation along the axial skeleton (Figures 1A,B, 3A,B, Supplementary Figure 9A, and Tables 1, 2). However, the subunit of PcG group protein Ezh2 inactivation only produced posterior transformation of L6 to S1 (Supplementary Figures 10–12 and Tables 1, 2). Furthermore, the phenotypes of double *Jmjd3* and *Ezh2* gene-deleted mice were similar to those of *Jmjd3* knockout mice (Figures 2A,B, Supplementary Figures 8A,B, 9A, and Table 2). Consistently, whole-mount *in situ* hybridization of mouse embryos at E9.5 showed that Jmjd3 was expressed more obviously in the head and trunk, while Ezh2 was expressed more strongly in the tail (Supplementary Figure 15). Therefore, these results strongly indicated that there is no continuous dynamic interplay between Jmjd3 and Ezh2 in the control of body axis patterning.

### Axial Bone Patterning May Synchronize With Chondrogenic Differentiation in Mice

Axial skeletal formation is primarily through the process of endochondral bone formation, which starts from the differentiation of condensed mesenchymal cells to cartilaginous templates (Karsenty, 2008; Long and Ornitz, 2013). *Hox* genes are well-known patterning genes that confer identity to skeletogenic condensations (Krumlauf, 1994). There are few studies with the conditional knockout mice to explore the mechanism of spatiotemporal control of *Hox* gene transcription. Here, chondrogenic cell conditional gene knockout mice were used to address the roles of Jmjd3 during the process of mouse body axial patterning. Previously, the transcription factor Sox9 of *Col2a1* was reported be observed in the mouse embryos at E8.0 (Zhao et al., 1997; Akiyama et al., 2005). Laz reporter gene of *Col2a1*-Cre mice (Ovchinnikov et al., 2000; Nakamura et al., 2006) and *in situ* hybridization with *Col2a1* probe experiments showed that *Col2a1* could be observed in mouse embryos at E8.5 (Ng et al., 1997). Consistently, the mRNA levels of *Col2a1* and *Sox9* could be detected by RT-qPCR in E8.5 embryos of mice (Supplementary Figure 1B). *Jmjd3* deletion in *Col2a1*-expressing chondrogenic cells by tamoxifen treatment at E8.5 could produce homeotic transformations of the whole-body axis (Figures 3A,B and Table 1). Therefore, we suggested that axial bone patterning may synchronize with chondrogenic differentiation. This idea is further supported by a previous report that the expression of retinoic acid receptor driven by the *Col2a1* promoter could induce homeotic transformations in mice (Yamaguchi et al., 1998).

In summary, we demonstrated that the temporal collinearity activation of *Hox* genes primarily relied on Jmjd3 to erasing H3K27me3 on *Hox* genes. Jmjd3 regulates not only the initiation but also the maintenance of the temporal collinear expression of *Hox* genes. No continuous dynamic interplay was detected between Jmjd3 and Ezh2 in the control of body axis patterning. Lastly, we demonstrated that chondrogenic cells were essential for axial skeletal patterning and proposed that axial bone patterning may synchronize with chondrogenic differentiation in mice.

## Materials and Methods

Materials, methods, and associated references are described in Supplementary Materials and Methods.

## Data Availability Statement

The authors declare that data supporting the findings of this study are available within the article and its Supplementary Material files or from the corresponding author on request. The RNA sequencing data cited in Supplementary Figures 1, 2 have been deposited in the NCBI SRA database under the accession code PRJNA673586.

## Ethics Statement

The animal study was reviewed and approved by Institutional Animal Care and Use Committee at the Fourth Military Medical University.

## Author Contributions

FZ conceived the project, performed most of the experiments, analyzed the data, and wrote the manuscript. XZ, RJ, YW, and XW help to raise the mice, collected the samples, and performed the molecular biology experiments. All the authors helped to analyze the data and approved the final manuscript.

## Conflict of Interest

The authors declare that the research was conducted in the absence of any commercial or financial relationships that could be construed as a potential conflict of interest.
