# Supplementary material for: Identification of Jmjd3 as an Essential Epigenetic Regulator of Hox Gene Temporal Collinear Activation for Body Axial Patterning in Mice
Source: Front Cell Dev Biol. 2021 Jul 21;9:642931. doi: 10.3389/fcell.2021.642931 (PMC8333871; doi:10.3389/fcell.2021.642931)
Supplement: Supplementary file 1 [file Data_Sheet_1.docx]

**Table 1 ^a^Homeotic transformations of axial skeletons with various genotypes**

| Genotypes | Time points | Total  (n) | Homeotic transformations of axial skeletons | | | | | | |
| --- | --- | --- | --- | --- | --- | --- | --- | --- | --- |
|  |  |  | ^b^C2 to C1 | ^c^T1 to C7 | ^d^T3 to T2 | ^e^T8 to T7 | ^f^L1 to T13 | ^g^S1 to L6 | ^h^L6 to S1 |
| ^i^Control |  | 60 |  |  |  |  |  |  | 4(6.7%) |
| *Jmjd3^fl/+^;Prx1-Cre* |  | 10 | 1(10.0%) |  |  |  | 7(70.0%) |  |  |
| *Jmjd3^fl/fl^;Prx1-Cre* |  | 14 | 14(100%) | 7(50.0%) | 3(21.4%) |  | 14(100%) | 10(100%) |  |
| *Utx^fl/Y^;Prx1-Cre* |  | 25 |  |  |  |  |  |  | 1(4.0%) |
| *Utx^fl/fl^;Prx1-Cre* |  | 22 |  |  |  |  |  |  | 2(9.0%) |
| *Ezh2^fl/+^;Prx1-Cre* |  | 17 |  |  |  |  |  |  | 10(58.8%) |
| *Ezh2^fl/fl^;Prx1-Cre* |  | 15 |  |  |  |  |  |  | 7(46.7%) |
| *Jmjd3^fl/fl^;Col2a1-Cre^ERT2^* | E8.5 | 18 | 7(38.9%) | 10(55.6%) | 6(33.3%) | 16(88.9%) | 18(100%) | 9(50%) |  |
|  | E9.5 | 17 |  |  |  | 15(88.2%) | 17(94.4%) | 13(76.5%) |  |
|  | E10.5 | 21 |  |  |  | 3(14.2%) | 4(19.0%) |  |  |
| *Ezh2^fl/fl^;Col2a1-Cre^ERT2^* | E8.5 | 20 |  |  |  |  |  |  | 12(60.0%) |
|  | E9.5 | 21 |  |  |  |  |  |  | 10(47.6%) |
|  | E10.5 | 16 |  |  |  |  |  |  | 7(43.8%) |
| *Jmjd3^fl/fl^;Ezh2^fl/fl^;*  *Col2a1-Cre^ERT2^* | E8.5 | 12 | 5(41.7%) | 8(66.7%) | 5(41.7%) | 10(83.3%) | 12(100%) | 9(75.0%) |  |
|  | E9.5 | 10 |  |  |  | 8(80.0%) | 9(90.0%) | 5(50.0%) |  |
|  | E10.5 | 25 |  |  |  | 4(16.0%) | 5(20.0%) | 3(12.0%) |  |

^a^Numbers of mice in each category are shown. Both unilateral and bilateral abnormalities were included in axial skeleton analysis. Number of sacral vertebrae was not counted because the cartilagiour linkages between the sacral vertebrae S3 and S4 are often only very fine at birth.^b^An extra ventral tubercle shared by fused C1 and C2. ^c^T1 without ribs or with incomplete ribs. ^d^Longest spinous process on T3 instead of T2. ^e^T8 with unilateral or bilateral vertebrosternal ribs. ^f^L1 with a pairs of complete or incomplete ribs. ^g^S1 without bilateral or unilateral ilial bones. ^h^L6 with bilateral or unilateral ilial bones. ^i^Mice of control group including the genotypes of *Col2a1-Cre^ERT2^, Jmjd3^fl/ fl^, Utx^fl/fl^ and Ezh2^fl/fl^, each* genotype contain 15 mice.

C, cervical; T, thoracic; L, lumbar; S, sacral; WT, Wild type.

**Table 2** **Skeletal analysis of E18.5 mouse embryos after treatment by GSK-J4 (50mg/kg) or GSK-126 (100mg/kg) at indicated time points^a^**

| Inhibitors | Vehicle | GSK-J4 | | | | | GSK-126 | | | | | |
| --- | --- | --- | --- | --- | --- | --- | --- | --- | --- | --- | --- | --- |
| **Time points** |  |  |  | **E8.5** | **E9.5** | **E10.5** | |  |  | **E8.5** | **E9.5** | **E10.5** |
| **Total number of mice** | **46** |  |  | **26** | **15** | **25** | |  |  | **29** | **17** | **16** |
| **Homeotic transformation of axial skeletons** |  |  |  |  |  |  | |  |  |  |  |  |
| **^b^T8 to T7** |  |  |  | **26(100%)** |  |  | |  |  |  |  |  |
| **^c^L1 to T13** |  |  |  |  | **15(100%)** |  | |  |  |  |  |  |
| **^d^S1 to L6** |  |  |  |  | **14(93.3%)** |  | |  |  |  |  |  |
| **^e^L6 to S1** | **2(4.3%)** |  |  | **1(3.8%)** | **0(0%)** | **1(4%)** | |  |  | **9(31.0%)** | **5(29.4%)** | **4(25.0%)** |

**^a^Numbers of mice in each category are shown and all mice were hybrid backgroud. Both unilateral and bilateral abnormalities were inclu**ded in axial skeleton analysis. Number of sacral vertebrae was not counted because the cartilagiour linkages between the sacral vertebrae S3 and S4 are often only very fine at birth.

^b^T8 with unilateral or bilateral vertebrosternal ribs;

^c^L1 with a pairs of complete or incomplete ribs;

^d^S1 without bilateral or unilateral ilial bones.

^e^L6 with bilateral or unilateral ilial bones.

C, cervical; T, thoracic; L, lumbar; S, sacral

**Supplementary Information**

**
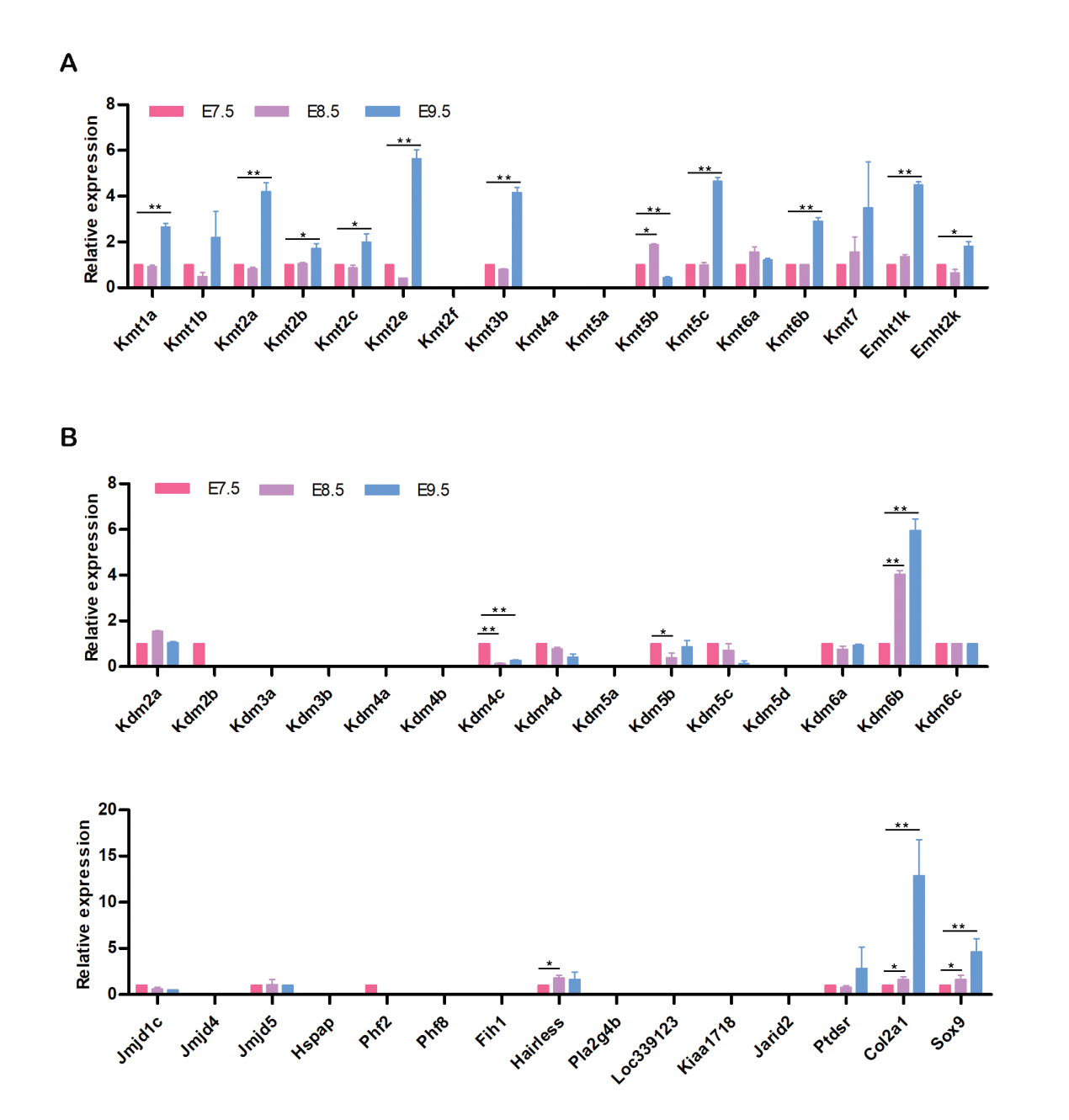
Figure S1. *Kdm6b* (*Jmjd3*) is significantly upregulated in E8.5 and E9.5 mouse embryos during the body axis development**

(A)The mRNA levels of 17 histone methyltransferase were screened by RT-qPCR in E7.5, E8.5 and E9.5 mouse embryos.

(B) The mRNA levels of 28 histone demethylase and two chondrogenic genes (*Col2a1* and *Sox9*) were examined by RT-qPCR in E7.5, E8.5 and E9.5 mouse embryos. Compared with E7.5 embryos, the mRNA level of *Kdm6b* (*Jmjd3*) was significantally increased in E8.5 and E9.5 mouse embryos respectively.

Bars indicate triplicate PCR reactions ± SD. Representative results of three independent experiments are shown. *p < 0.05, ** p < 0.01.

**
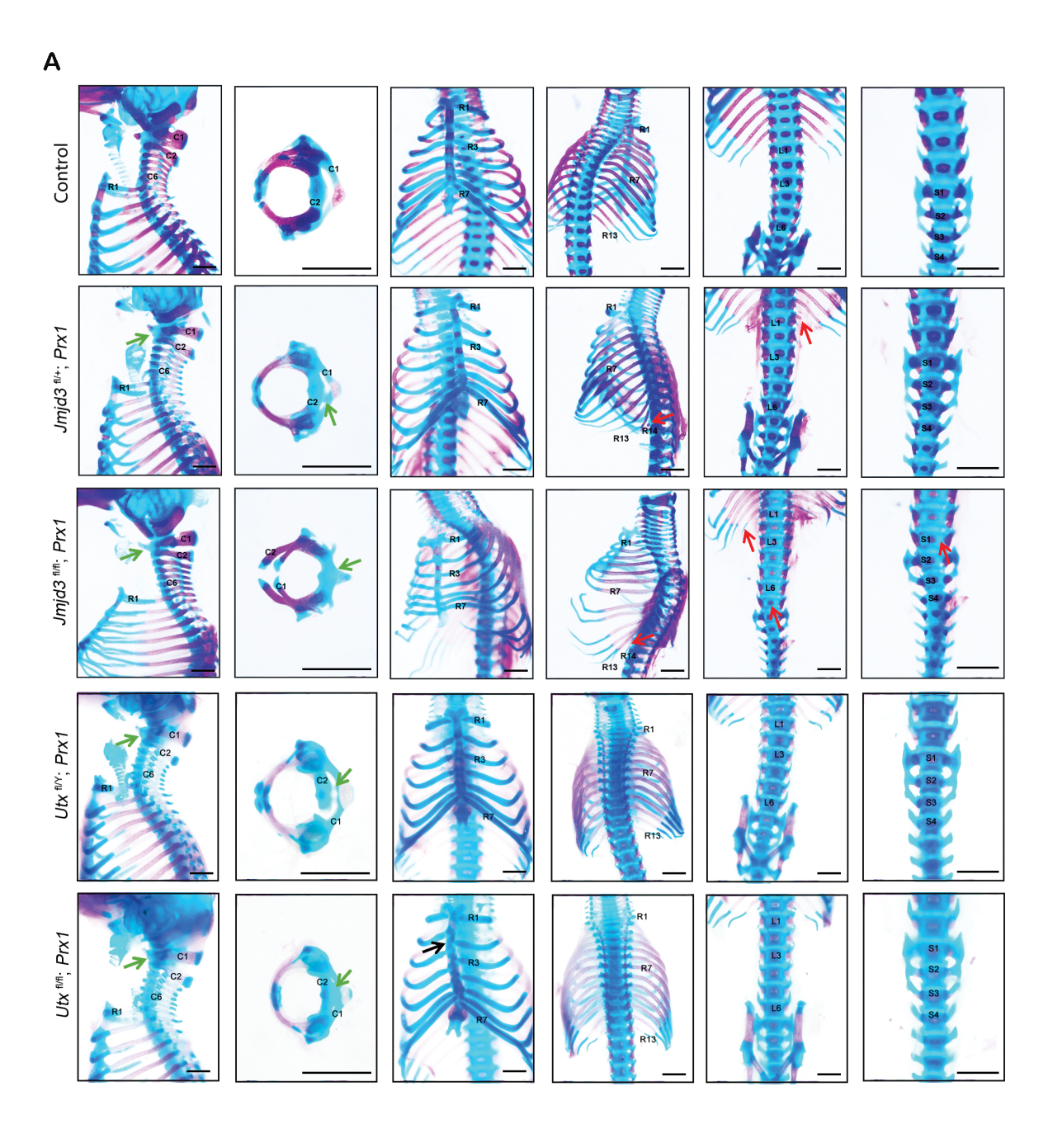
Figure S2. Homeotic transformations and skeletal abnormalities in E18.5 *Jmjd3*^fl/+^;*Prx1*, *Jmjd3*^fl/fl^;*Prx1*, *Utx*^fl/Y^;*Prx1* and *Utx*^fl/fl^;*Prx1* mouse embryos.**

(A) Skeletal phenotypes were prepared from E18.5 mouse embryos with various genotypes as indicated. The red arrows indicate homeotic transformations of axial skeleton, the green arrows indicate fusions of C1 and C2 and black arrow indicated sternal asymmetries in *Utx*^fl/fl^;*Prx1* mice. Frequencies of homeotic transformations are given in Tables 1. The cervical (C), ribs (R), lumbar (L), and sacral (S) vertebrae are numbered. Scale bar, 1 mm.

**
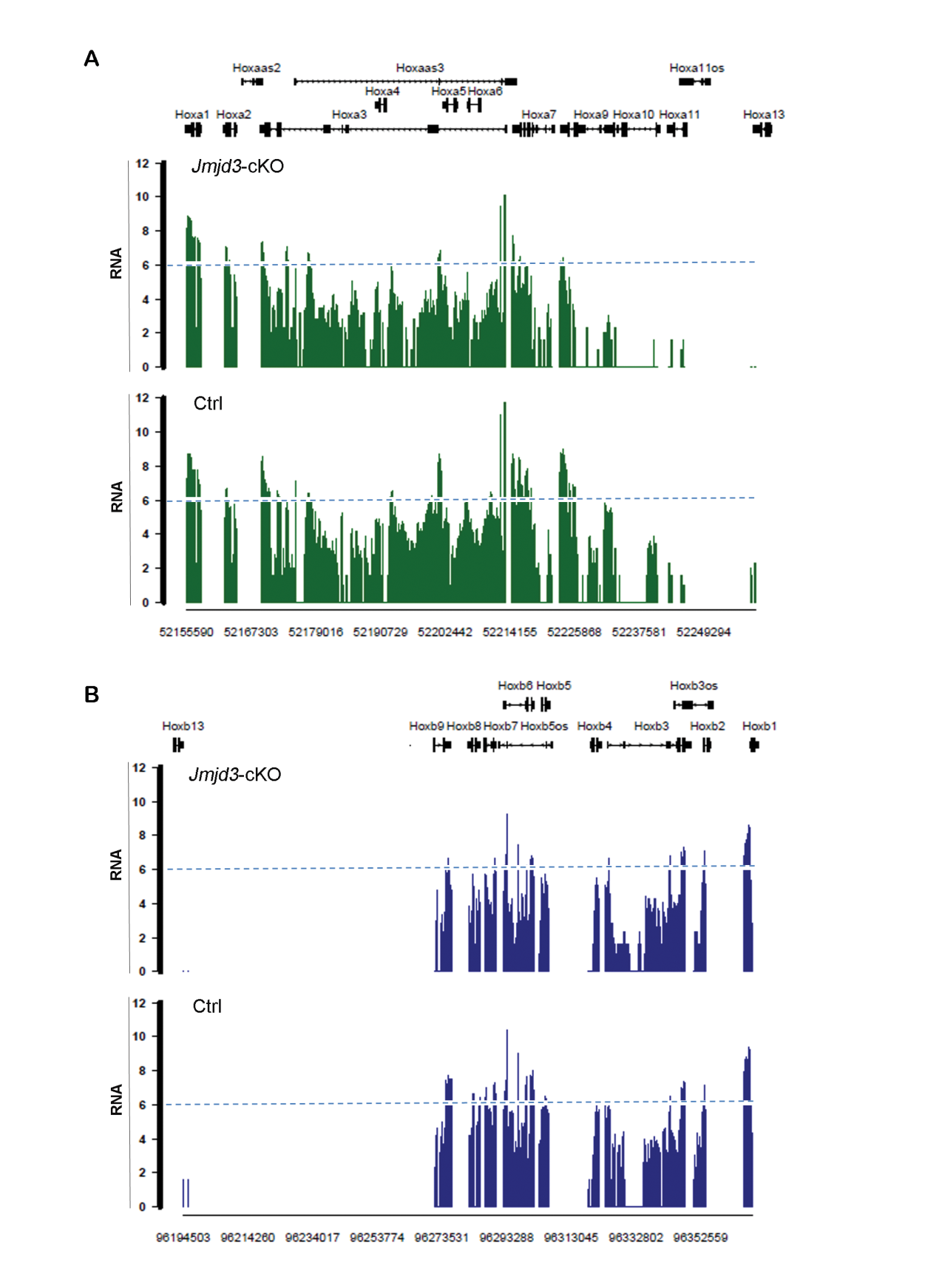
Figure S3 Jmjd3 loss in uncommitted mesenchymal cells affected the expression of HoxA and HoxB clusters.**

(A,B) RNA-seq assays indicated that *Jmjd3* loss in uncommitted mesenchymal cells mildly decreased the expression of *Hoxa3~5*(A)*, while* markedly reduced the mRNA level of *Hoxa6~10* and *Hoxb6~9* compared to WT in E8.5 littermates. The y-axis represents the mRNA transcription level. The higher the signal peak, the higher the mRNA transcription level of Hox gene. The lower the signal peak, the lower the mRNA transcription level of Hox gene.

**
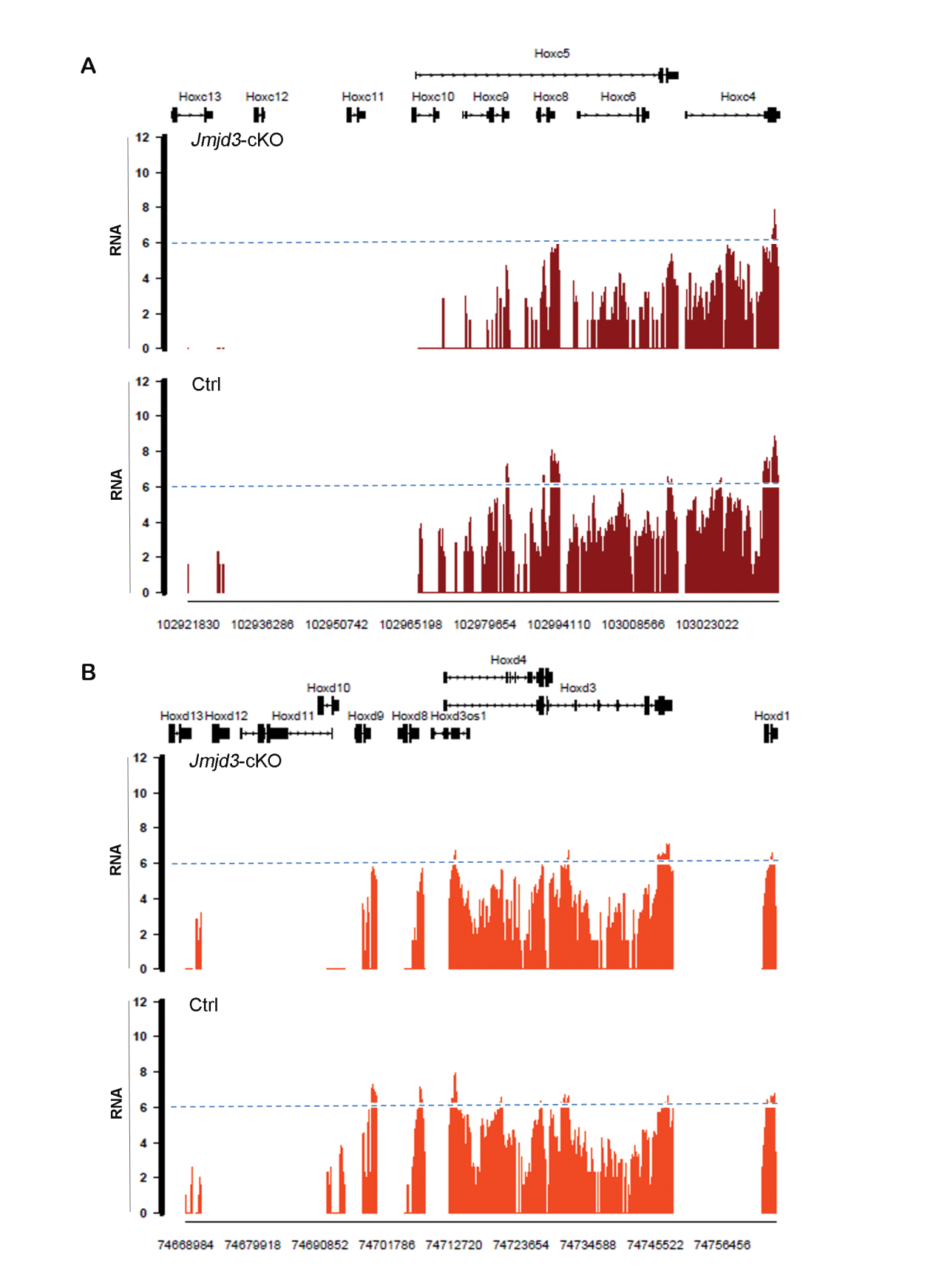
Figure S4 Jmjd3 loss in uncommitted mesenchymal cells affected the expression of HoxC and HoxD clusters.**

(A,B) RNA-seq assays indicated that Jmjd3 loss in uncommitted mesenchymal cells markedly reduced the mRNA level of *Hoxc4~c10* (A) and *Hoxd8~d10*(B) compared to WT control in E8.5 littermates. The y-axis represents the mRNA transcription level. The higher the signal peak, the higher the mRNA transcription level of Hox gene. The lower the signal peak, the lower the mRNA transcription level of Hox gene.

**
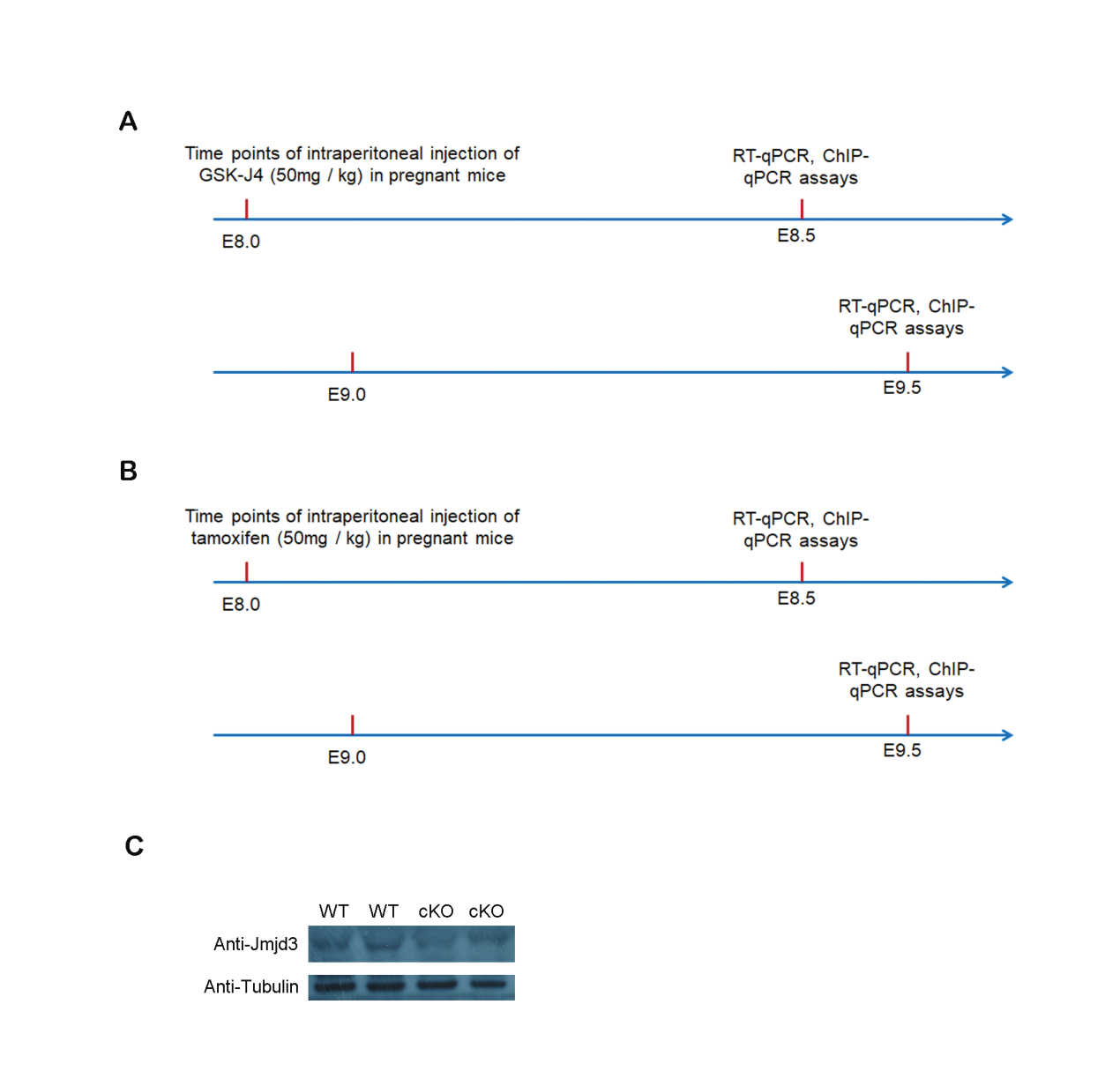
Figure S5 Schematic representation of experimental design for studying the roles of Jmjd3 on *Hox* gene expresion.**

(A) Experimental design for studying the effects of GSK-J4 on *Hox* gene expresion.

(B) Experimental design for studying the roles of Jmjd3 in chondrogenic cells on *Hox* gene expresion.

(C) Compared with WT mice, western blot detected the protein level of Jmjd3 decreased in *Jmjd3*^fl/fl^;*Col2a1*^ERT2^ embryos 12 hours later after tamoxifen (50mg/kg) treatment.

**
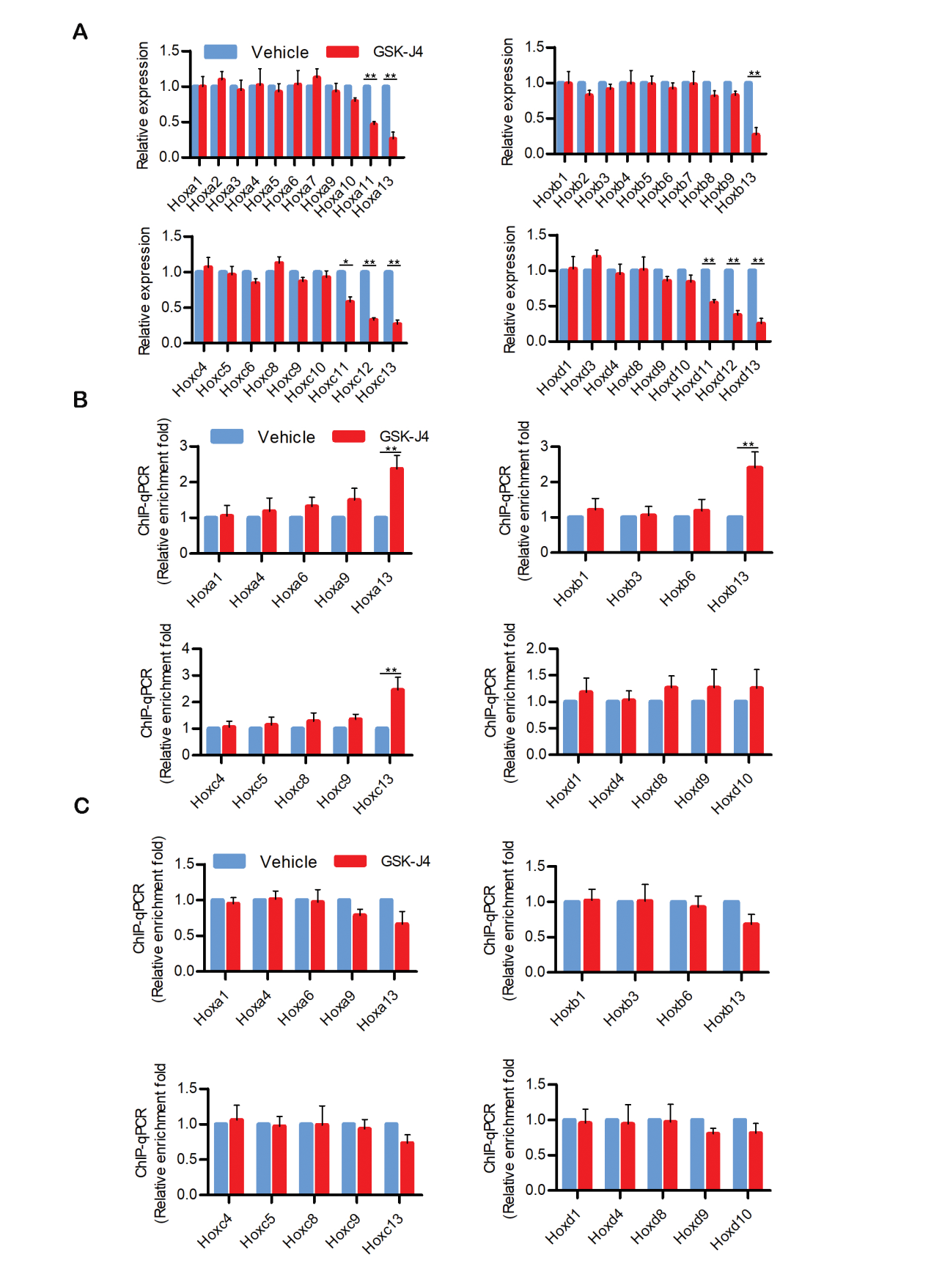
Figure S6. GSK-J4 inhibits the transcription of *Hox* genes by block H3K27me3 demethylase activity of Jmjd3 in E9.5 mouse embryos.**

(A) The mRNA levels of *Hox* genes were determined by RT-qPCR in E9.5 embryos treated by vehicle or GSK-J4 (50mg/kg) 12 hours before.

(B,C) ChIP-qPCR assays with antibodies specific for H3K27me3 (B) or Jmjd3 (C) on the *Hox* genes in E9.5 embryos treated by vehicle or GSK-J4 (50mg/kg) 12 hours before. Bars indicate triplicate PCR reactions ± SD. Representative results of three independent experiments are shown. *p < 0.05, ** p < 0.01.

**
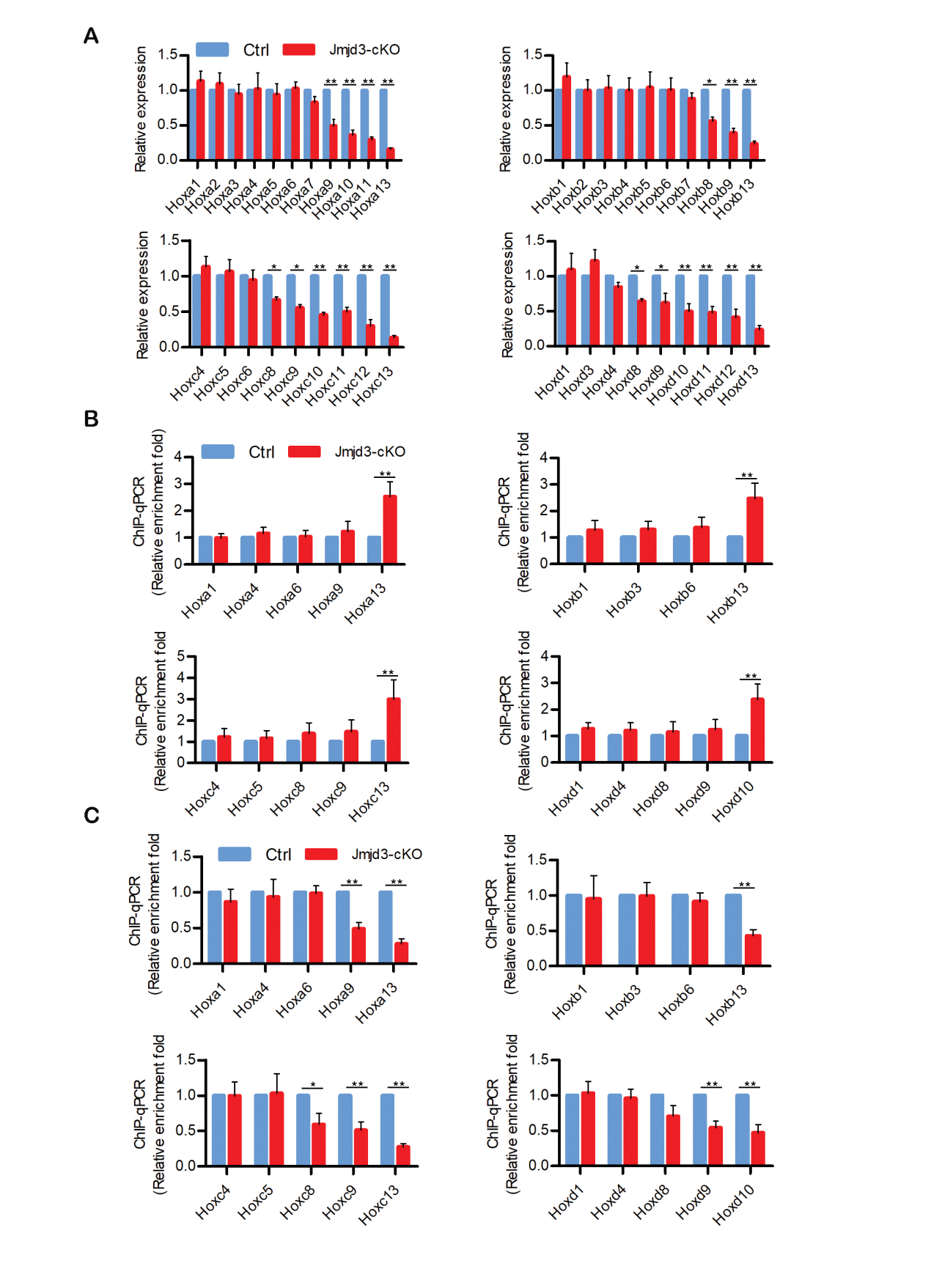
Figure S7. Jmjd3 temporally activated and maintained *Hox* gene expression in E9.5 mouse embryos.**

(A) The expression levels of *Hox* genes were determined by RT-qPCR in control or *Jmjd3*-cKO E9.5 embryos treated by tamoxifen (50mg/kg) 12 hours before.

(B,C) ChIP-qPCR assays with antibodies specific for H3K27me3 (B) or Jmjd3 (C) on the *Hox* genes in control or *Jmjd3*-cKO E9.5 embryos treated by tamoxifen (50mg/kg) 12 hours before.

Bars indicate triplicate PCR reactions ± SD. Representative results of three independent experiments are shown. *p < 0.05, ** p < 0.01.

**
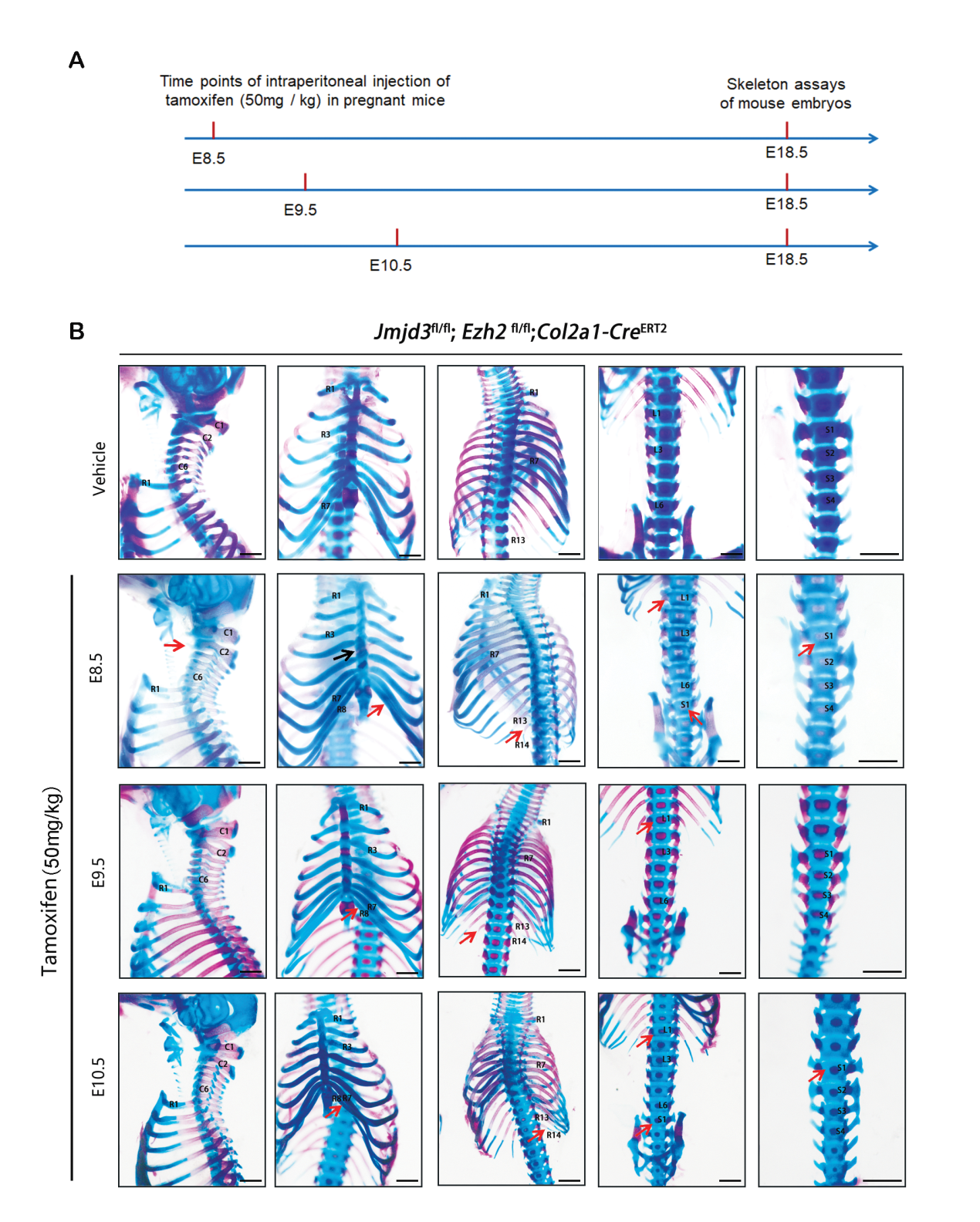
Figure S8. Homeotic transformations by Jmjd3 and Ezh2 conditional double knockout at E8.5, E9.5 and E10.5 days respectively**

(A) Experimental design for studying the roles of Jmjd3 and Ezh2 in chondrogenic cells on body axis patterning.

(B) Skeletal phenotypes were prepared from E18.5 embryos of mice.

The red arrow indicates homeotic transformations of axial skeletons and black arrow showed sternal asymmetries. Frequencies of homeotic transformations are given in Tables 1. For schematic explanation see Figure 8. The cervical (C), ribs (R), lumbar (L), and sacral (S) vertebrae are numbered. Scale bar, 1 mm.

**
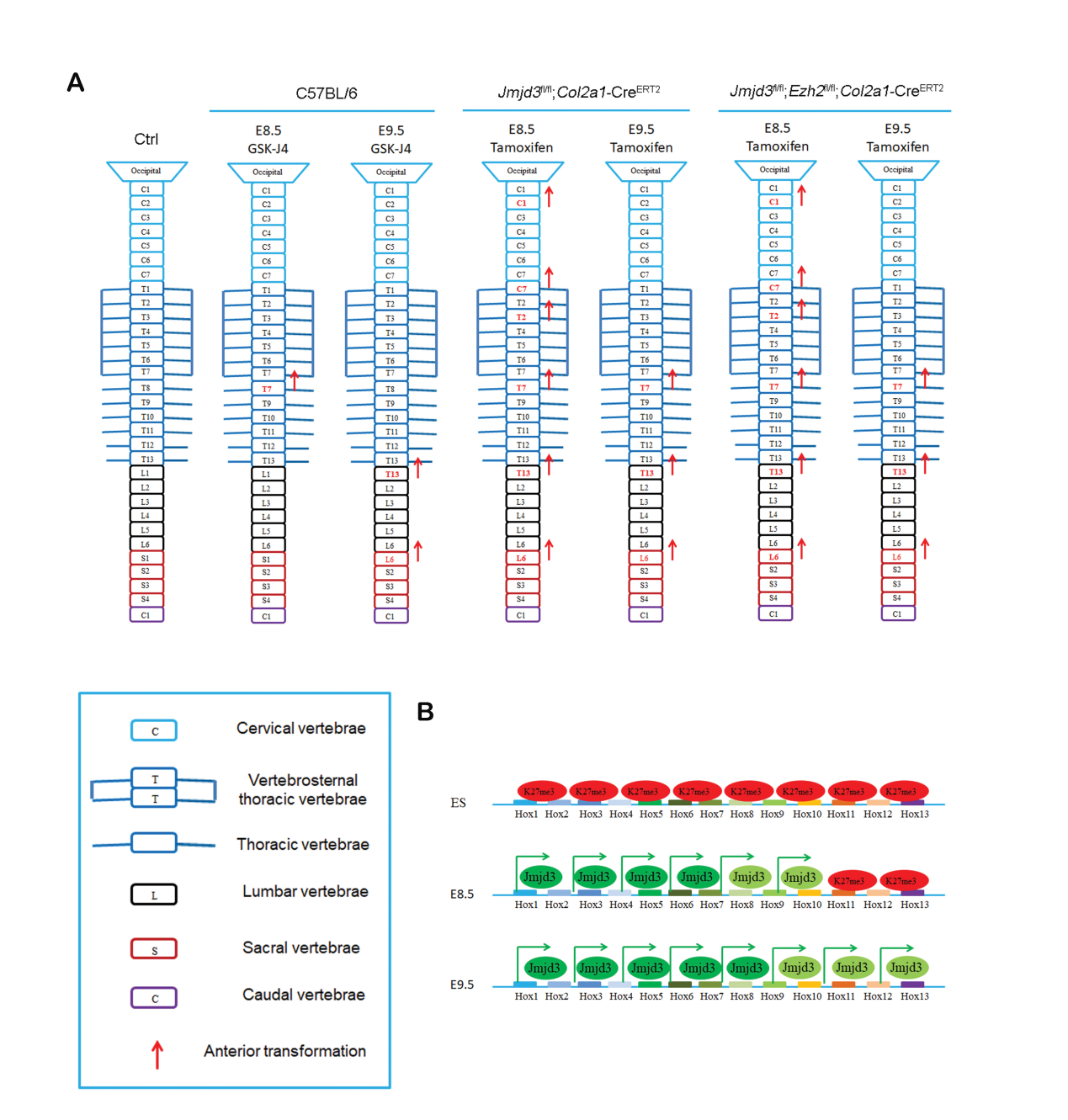
Figure S9 Schematic representation of Jmjd3 as an essential epigenetic regulator of *Hox* gene temporal collinear activation for body axial patterning in mice**

A) Each group of vertebrae is represented with a colour code: cervical (C), thoracic (T), lumbar (L), sacral (S) and caudal (C). Anterior transformations are identified with red arrows and the affected segments are marked by red colour letter. See text for references and details.

B) Each Hox cluster is scaled on the basis of coordinates with the gene name listed below. Arrows indicate the direction of transcription. Expression of *Hox* gene along the anterior-to-posterior (AP) embryonic axis is collinear with gene order within the cluster. In embryonic stem (ES) cells, the whole cluster is labelled with H3K27me3 (red). During axial extension, the sequential onset of *Hox* gene transcriptional activation is accompanied with progressive demethylation of H3K27me3 by Jmjd3. Light green Jmjd3 indicates activation of *Hox* gene expression. Green Jmjd3 indicates maintenance of *Hox* gene expression.

**
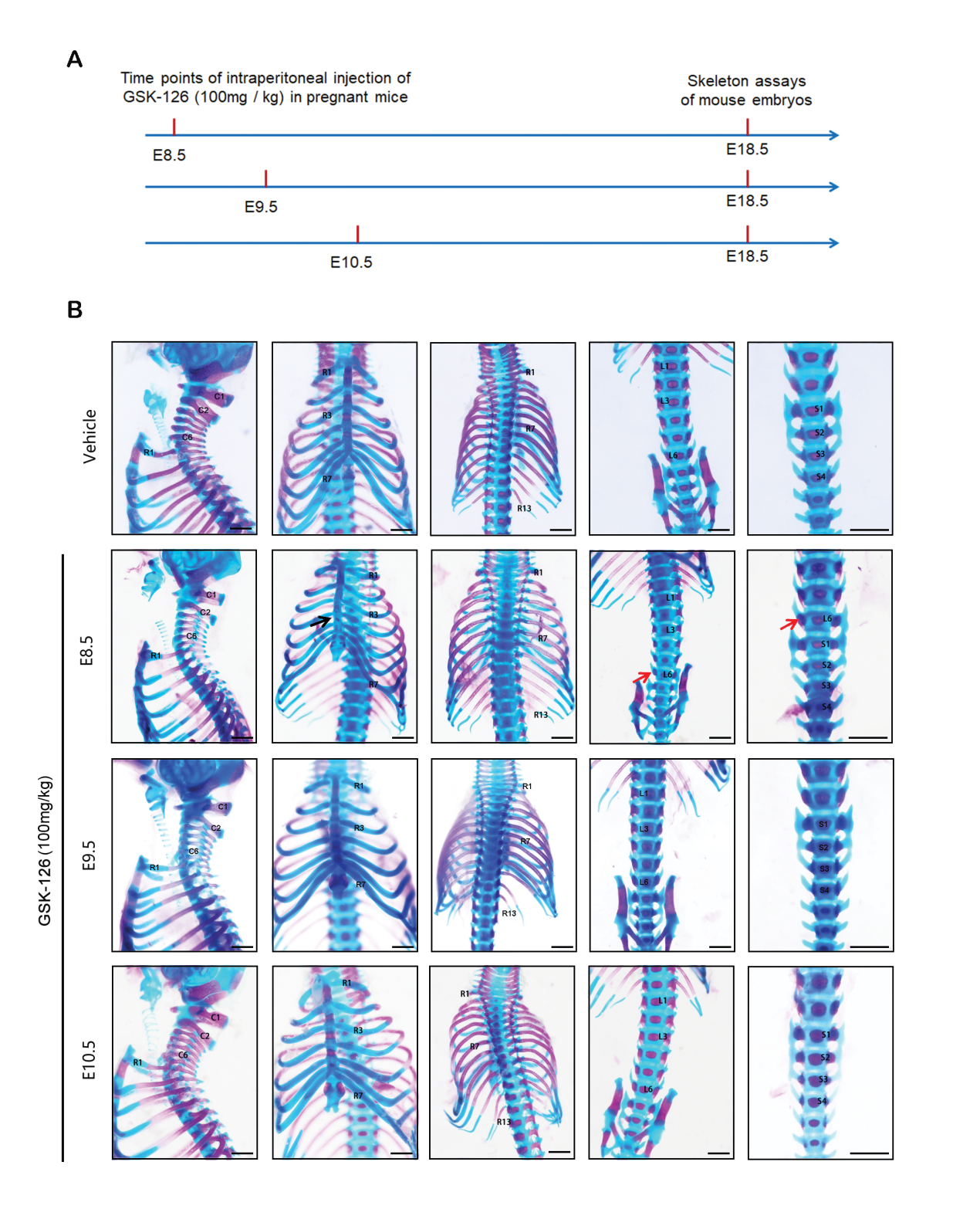
Figure S10. Homeotic transformations by GSK-126 exposure at E8.5, E9.5 and E10.5 days.**

(A) Experimental design for studying the roles of GSK-126 on body axis patterning.

(B) Skeletal phenotypes were prepared by removing forelimbs and hindlimbs.

The red arrow indicates homeotic transformations of axial skeleton and black arrow showed sternal abnormalities induced by GSK-126 at the time point as indicated. For schematic explanation see Figure 8.Frequencies of homeotic transformations are given in Tables 2. The cervical (C), ribs (R), lumbar (L), and sacral (S) vertebrae are numbered. Scale bar, 1 mm.

**
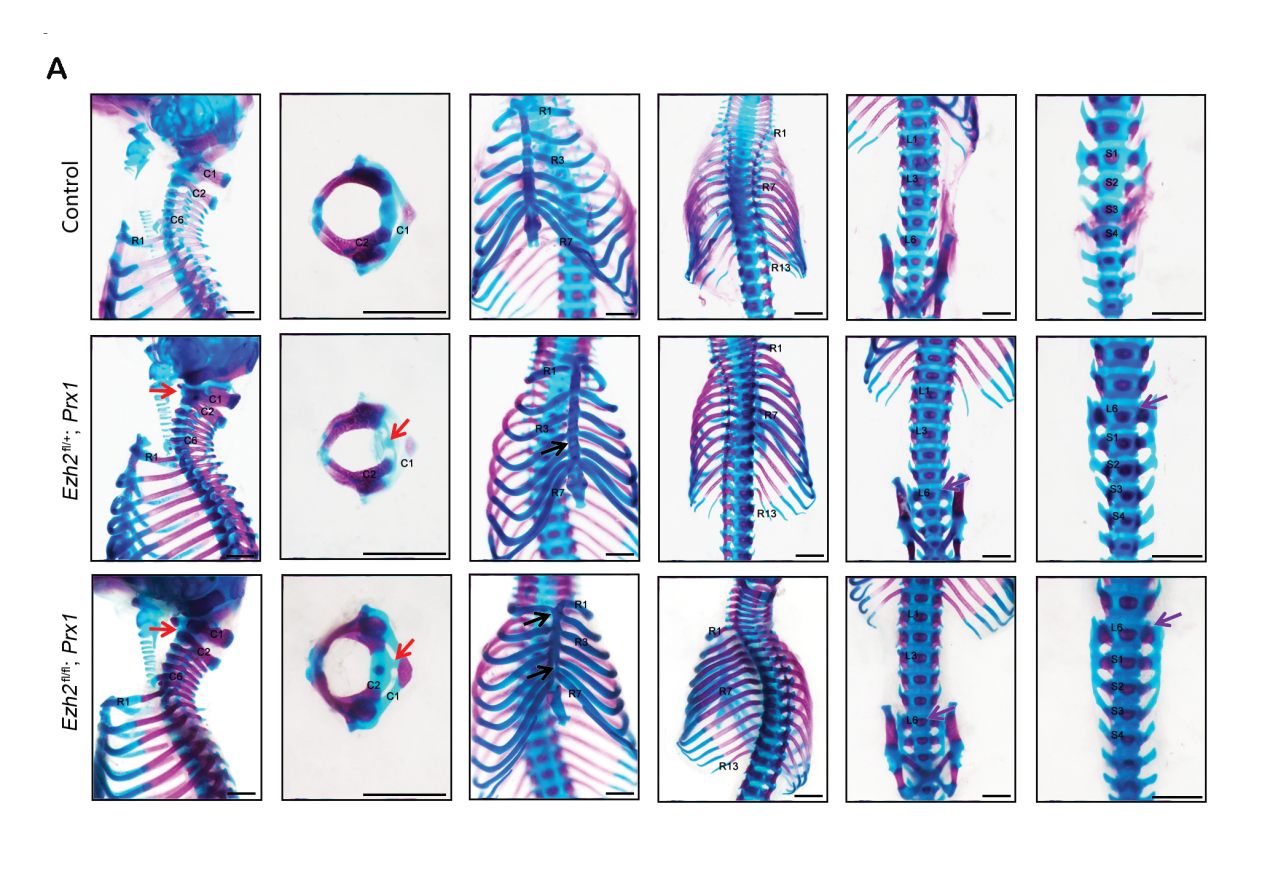
Figure S11. Homeotic transformations and skeletal abnormalities in *Ezh2*^fl/+^;*Prx1* and *Ezh2*^fl/fl^;*Prx1* mice.**

(A)Skeletal phenotypes were prepared from E18.5 embryos of mice. The red arrows indicate fusions of C1 and C2 and the violet arrows indicated homeotic transformations of axial skeleton in *Ezh2*^fl/+^;*Prx1* and *Ezh2*^fl/fl^;*Prx1* mice. The black arrows indicate sternal asymmetries. Frequencies of homeotic transformations are given in Tables 1. The cervical (C), ribs (R), lumbar (L), and sacral (S) vertebrae are numbered. Scale bar, 1 mm.

**
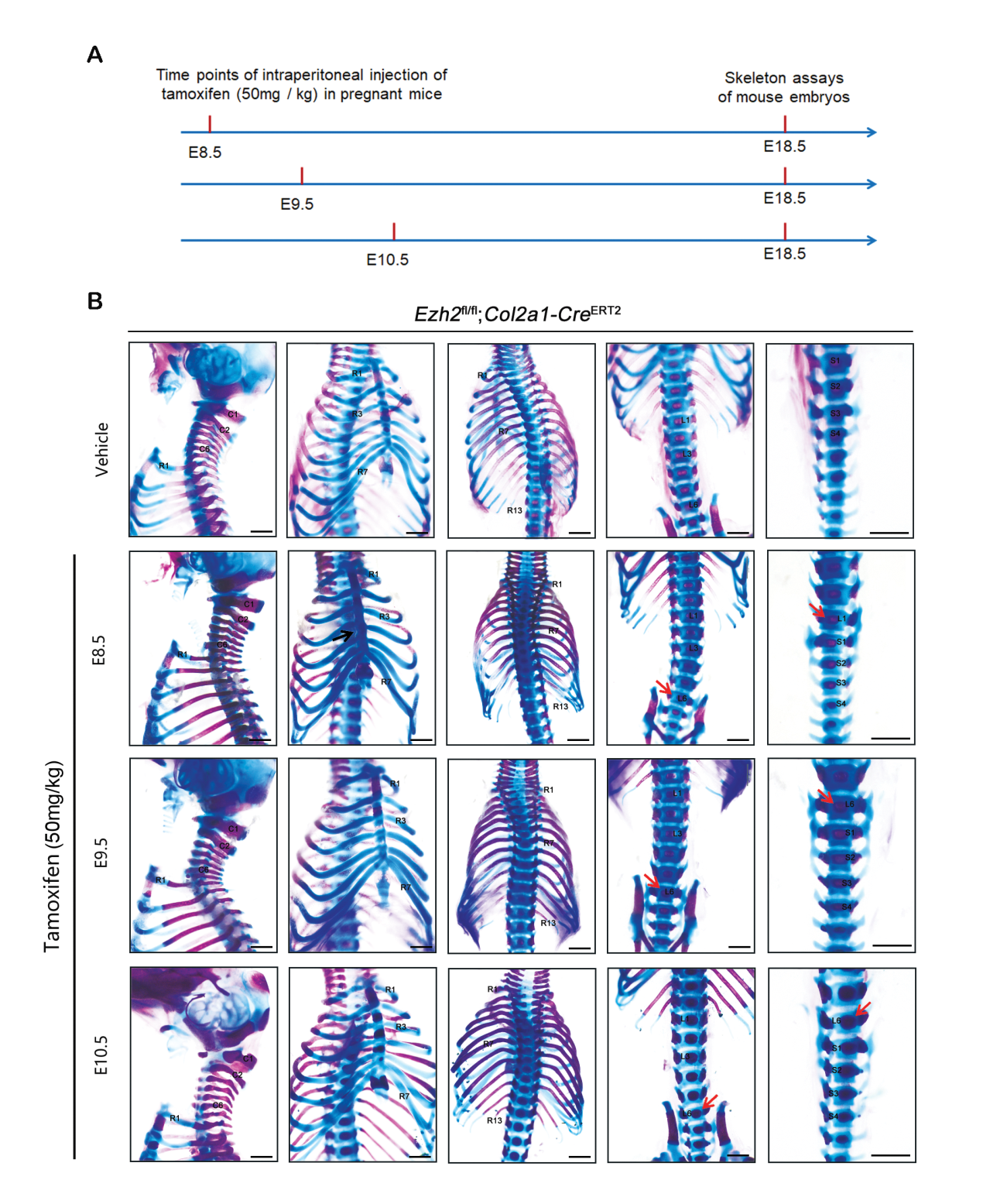
Figure S12. Homeotic transformations by Ezh2 conditional knockout in *Col2a1* expressed chondrogenic cells at E8.5, E9.5 and E10.5 days.**

(A) Experimental design for studying the roles of Ezh2 in chondrogenic cells on body axis patterning.

(B) Skeletal phenotypes were prepared from E18.5 embryos of mice.

The red arrow indicates homeotic transformations of axial skeletons and black arrow indicated sternal asymmetries.Frequencies of homeotic transformations are given in Tables 1. The cervical (C), ribs (R), lumbar (L), and sacral (S) vertebrae are numbered. For schematic explanation see Figure 8. Scale bar, 1 mm.


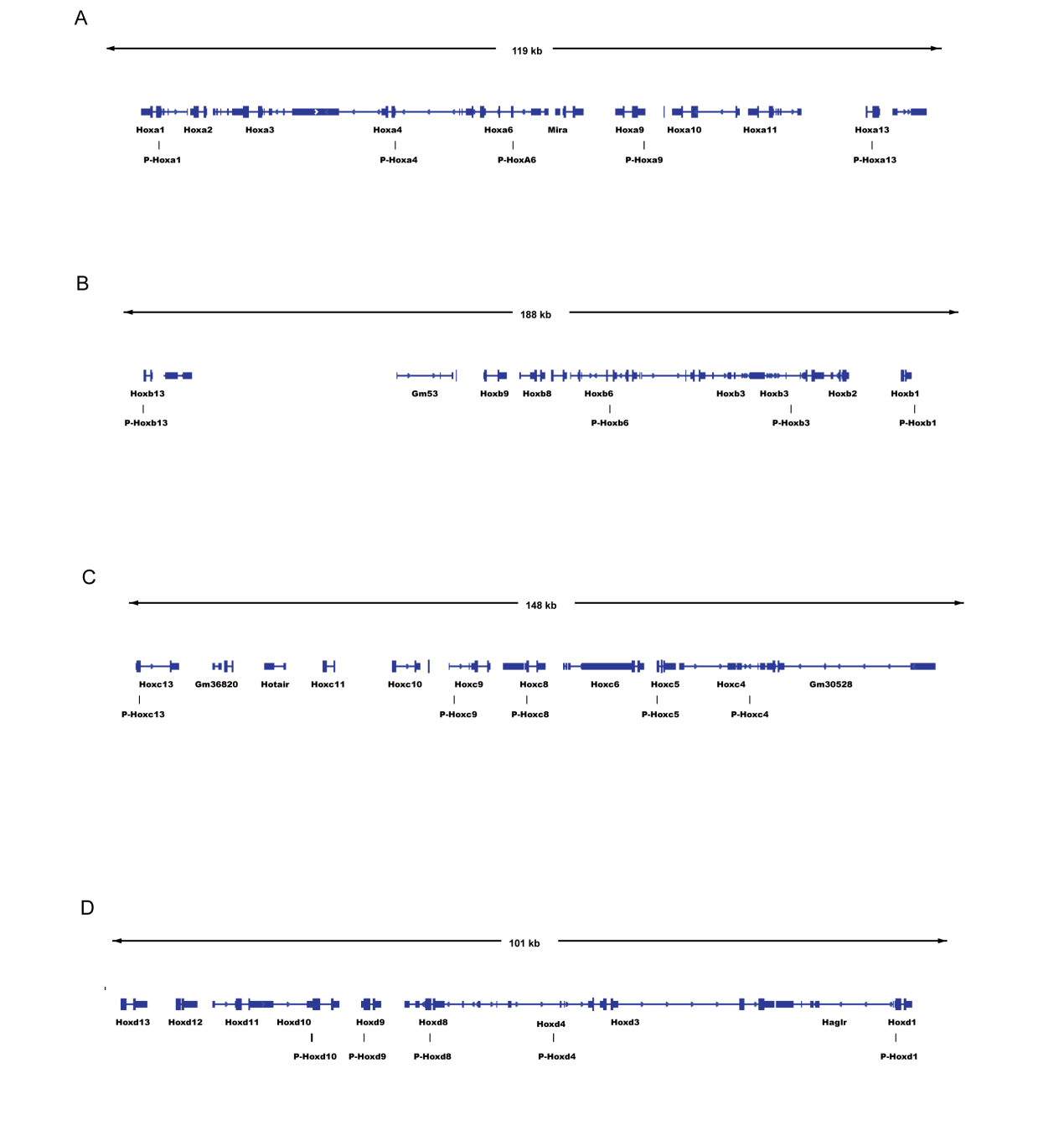


**Figure S13 ChIP-qPCR assays at the Hoxa, Hoxb, Hoxc and Hoxd loci.**

A-D) Positions of DNA fragments amplified by PCR for ChIP-qPCR assays at Hoxa (A) Hoxb (B), Hoxc (C) and Hoxd (D) locus.

**
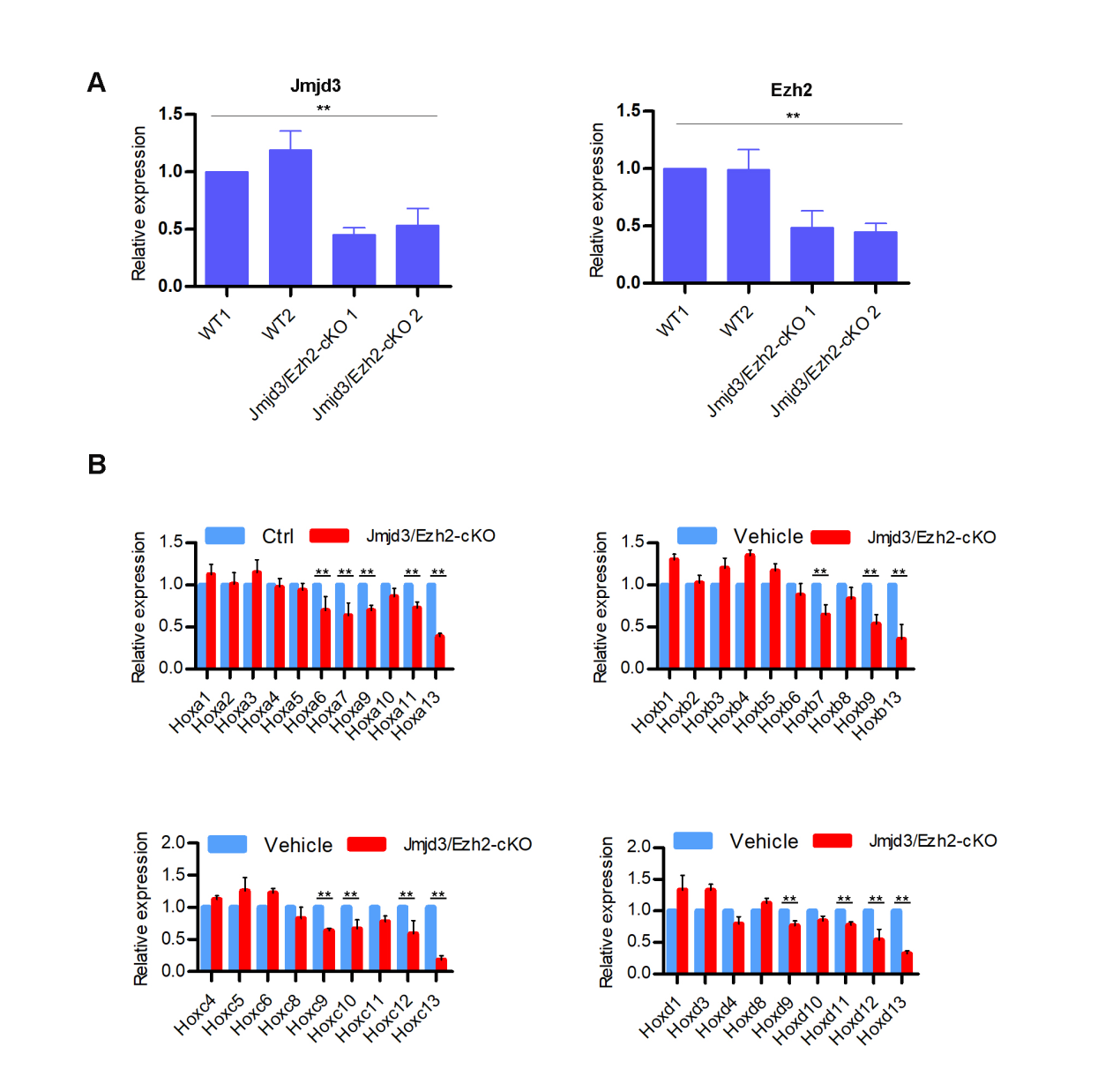
**

**Figure S14. Jmjd3 instead of Ezh2 temporally activated and maintained *Hox* gene expression in E9.5 mouse embryos.**

(A) The expression levels of *Jmjd3* or *Ezh2* genes were determined by RT-qPCR in control or *Jmjd3/Ezh2*-cKO E9.5 embryos treated by tamoxifen (50mg/kg) 12 hours before. ** p < 0.01.

(B) The expression levels of *Hox* genes were determined by RT-qPCR in control or *Jmjd3/Ezh2*-cKO E9.5 embryos treated by tamoxifen (50mg/kg) 12 hours before. ** p < 0.01.

**
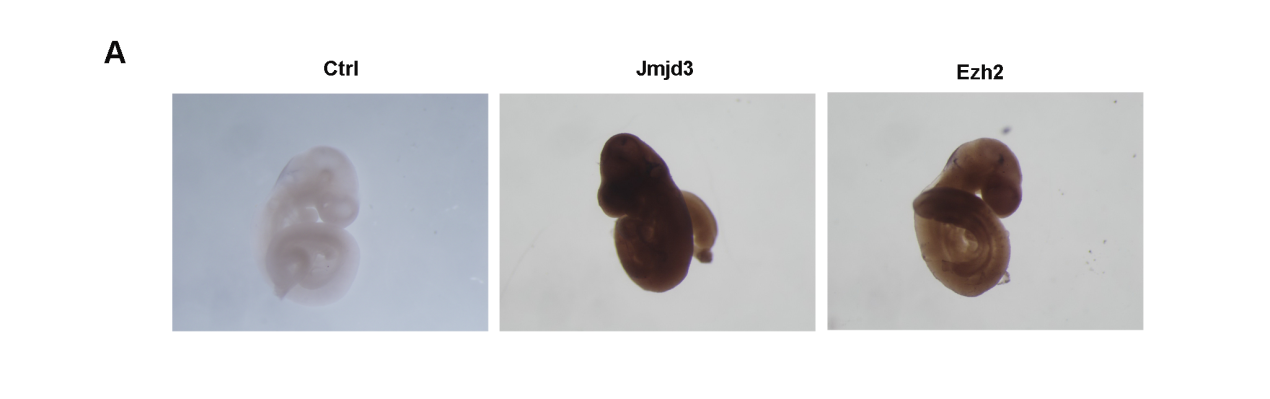
**

**Figure S15. Whole-mount in situ hybridization of mouse embryos at E9.5.**

(A), *Jmjd3* and *Ezh2* expression patterns of E9.5 embryos are shown by whole-mount in situ hybridization with *Jmjd3* or Ezh2 probes. Jmjd3 was expressed more obviously in the head and trunk, while Ezh2 was expressed more strongly in the tail.

**Supplementary Table 1. The PCR primers used for genotypes**

| **Strains** |  | **Primer sequences** | **Sizes** |
| --- | --- | --- | --- |
| ***Jmjd3-floxed*** | Forward  Reverse | GAGACCCCTGTTTGGTTACCAG  GAGGCAGAACCTGAGCAAGACC | 508bp |
| ***Ezh2-floxed*** | Forward  Reverse | CAT GTG CAG CTT TCT GTT CA  CAC AGC CTT TCT GCT CAC TG | 300bp |
| ***Utx-floxed*** | Forward  Reverse | TGGCAGGCATTAAACCTGAATC  ACATAGCCCATGAATGCTCTGC | 352bp |
| ***Col2a1-Cre^ERT2^*** | Forward  Reverse | CAC TGC GGG CTC TAC TTC AT  ACC AGC AGC ACT TTT GGA AG | 358bp |
| ***Prx1-Cre*** | Forward  Reverse | TCTCTGGCTCTGATGTTGGCA  CGCATAACCAGTGAAACAGC | 750bp |

**Supplementary Table 2. The FPKM of Hox genes in E8.5 WT and Jmjd3-KO mouse embryos by RNA-seq assays.**

| Gene name | FPKM(WT) | FPKM(Jmjd3-KO^a^) |  | Gene name | FPKM(WT) | FPKM(Jmjd3-KO) |
| --- | --- | --- | --- | --- | --- | --- |
| Hoxa1 | 22.74179525 | 24.50564718 |  | Hoxb1 | 41.3633346 | 27.04427233 |
| Hoxa2 | 7.682042027 | 9.880508431 |  | Hoxb2 | 2.603101483 | 1.744532211 |
| Hoxa3 | 21.76422617 | 7.010052509 |  | Hoxb3 | 4.282703658 | 4.757975344 |
| Hoxa4 | 2.678666335 | 1.640678555 |  | Hoxb4 | 4.723521122 | 2.398139797 |
| Hoxa5 | 3.522243875 | 2.622767466 |  | Hoxb5 | 4.443035784 | 2.826403957 |
| Hoxa6 | 4.570061837 | 1.584152668 |  | Hoxb6 | 16.35193613 | 6.275408166 |
| Hoxa7 | 14.56225727 | 4.682827145 |  | Hoxb7 | 15.17471743 | 7.502173802 |
|  |  |  |  | Hoxb8 | 1.95434579 | 1.663134888 |
| Hoxa9 | 10.57031168 | 1.890708742 |  | Hoxb9 | 16.31119623 | 5.787098217 |
| Hoxa10 | 0.897021551 | 0.091093693 |  |  |  |  |
| Hoxa11 | 0.094245638 | 0.070174343 |  |  |  |  |
|  |  |  |  |  |  |  |
| Hoxa13 | 0.04707128 | 0 |  | hoxb13 | 0 | 0 |
| (Continue) |  |  |  |  |  |  |
| Gene name | FPKM(WT) | FPKM(Jmjd3-KO) |  | Gene name | FPKM(WT) | FPKM(Jmjd3-KO) |
|  |  |  |  | Hoxd1 | 12.50108225 | 7.683797991 |
|  |  |  |  |  |  |  |
|  |  |  |  | Hoxd3 | 2.867509768 | 4.463234471 |
| Hoxc4 | 22.2172817 | 8.0969015 |  | Hoxd4 | 1.826712875 | 0.641251962 |
| Hoxc5 | 3.970856903 | 2.521968791 |  |  |  |  |
| Hoxc6 | 0.730928884 | 0.66334276 |  |  |  |  |
|  |  |  |  |  |  |  |
| Hoxc8 | 12.62532698 | 2.707543694 |  | Hoxd8 | 2.031624903 | 1.740791449 |
| Hoxc9 | 4.807850005 | 0.306840987 |  | Hoxd9 | 14.18604307 | 3.745200324 |
| Hoxc10 | 0.649374195 | 0 |  | Hoxd10 | 0.682629592 | 0 |
| Hoxc11 | 0 | 0 |  | Hoxd11 | 0 | 0 |
| Hoxc12 | 0 | 0 |  | Hoxd12 | 0 | 0 |
| Hoxc13 | 0.167926503 | 0 |  | Hoxd13 | 0.332818452 | 0 |

^a^Jmjd3 loss in uncommitted mesenchymal cells in E8.5 mouse littermates

**Supplementary Table 3. The PCR primers used for RT-qPCR**

| **Genes for histone methyltransferases** |  |
| --- | --- |
| Kmt1a-F | CCTGCACAAGTTTGCCTACA |
| Kmt1a-R | AGTGCGGAAGATGCAGAGAT |
| Kmt1b-F | ATCCCACCTGGTACTCCCATCT |
| Kmt1b-R | GCAAAGCGAATACTGTGTGCC |
| Kmt1c(Ehmt2)-F | GC TCCACCTGTCTACATCAT |
| Kmt1c(Ehmt2)-R | GCAGATGTTTTCCTCA TTGT |
| Kmt1d(Ehmt1)-F | CATAGCAAAAGCAGACACAA |
| Kmt1d(Ehmt1)-R | ACTTTCCAAGGTTTCCTTTC |
| Kmt2a-F | GCAGATTGTAAGACGGCGAG |
| Kmt2a-R | GAGAGGGGGTGTTCCTTCCTT |
| Kmt2c-F | GTGGCTGTTCCACACCCAG |
| Kmt2c-R | AGCTTGAGCTTCTCAGCATCG |
| Kmt2c-F | TGTTCACAGTGTGGTCAATGTT |
| Kmt2c-R | GAGGGTCTAGGCAGTAGGTATG |
| Kmt2e-F | TTCAGACCTCCTGTAGAGAGC |
| Kmt2e-R | AAAAGTCCTCGCATCAACACA |
| Kmt2f-F | GCCACGCAGTGAGTTTGA |
| Kmt2f-R | ACCCAGTGAGTGTCGTTGAG |
| Kmt3b-F | TCCGGTGAATTTAGATGCCTCC |
| Kmt3b-R | CGGTAACTGCATAGTACACCCAT |
| Kmt4a-F | GTTCTACCAGCTACCTCCGAGCGTGCAGC |
| Kmt4a-R | GCTGCACGCTCGGAGGTAGCTGGTACAAC |
| Kmt5a-F | AAGGTGGACTTGAACAGATG |
| Kmt5a-R | ACCTGTGCTGAGTCTTTGAC |
| Kmt5b-F | CGGCTGCTTCCAACTCTACC |
| Kmt5b-R | AGTGATTCCGCAGTCTGATCT |
| Kmt5c-F | GCAGAGCTGCGTGAAGAGG |
| Kmt5c-R | ACAGGCAGTATTCCCATCTGA |
| Kmt6a(Ezh1)-F | GGGAGCAAAGGCTCTGTATGTG |
| Kmt6a(Ezh1)-R | AGTTTCTTCCACTCTTCATTGAG |
| Kdm6b(Ezh2)-F | AACCGCTTTCCTGGATGTCG |
| Kdm6b(Ezh2)-R | CAATGGTCAGCAGCTCCACA |
| Kmt7-F | ATGGATAGCGACGACGAGGTA |
| Kmt7-R | CGAAGTTGCCCTCAAATCTGT |
|  |  |
| **Genes for histone demethylases** |  |
| Kdm2a-F | CGGATAGTTGAGAAAGCCAAGATCCG |
| Kdm2a-R | CTCTTTGGTGGGCCTCTGTAGC |
| Kdm2b-F | GTTAGTGGTAGTGGTGTTTTGG |
| Kdm2b-R | AGCAGATGTGGTGTGTGGTC |
| Kdm3a-F | ACCTGCAGTTATTCTTCAGC |
| Kdm3a-R | TAATGCCAGTCCTATGCCAT |
| Kdm3b-F | TGTTCCCTGGGGACTCCTCT |
| Kdm3b-R | GGGCACTACAGTACAGCTGG |
| Kdm4a-F | CCTCACTGCGCTGTCTGTAT |
| Kdm4a-R | CCAGTCGAAGTGAAGCACAT |
| Kdm4b-F | CGGGTTCTATCTTTGTTTCTCTCACCCG |
| Kdm4b-R | AAGGAAGCCTCTGGAACACCTG |
| Kdm4c-F | GGCATAGGTGACAGGGTGTGTC |
| Kdm4c-R | CGGGGACCAAACTCTGGAAACCCG |
| Kdm4d-F | CGGGATCTGCACAGATTATCCACCCG |
| Kdm4d-R | AGTTTCTGAGGAGGGCGACCA |
| Kdm5a-F | GTTTCTTAAGGTGGCAAGTC |
| Kdm5a-R | TCTTTTGTACTGTTCCCTAC |
| Kdm5b-F | AGCTTTCTCAGAATGTTGGC |
| Kdm5b-R | GCAGAGTCTGGGAATTCACA |
| Kdm5c-F | GGGTTTCTAAAGTGTAGATCT |
| Kdm5c-R | CCACACATCTGAGCTTTAGT |
| Kdm5d-F | ATCTCCTCACCTCTCCAAAG |
| Kdm5d-R | TTGTCTCTAGGCGTGGCCGT |
| Kdm6a(Utx)-F | CGTCCGAGTGTCAACCAACTGGACG |
| Kdm6a(Utx)-R | TGAGAGTCCTGGAGTAGGAGCAG |
| Kdm6b(Jmjd3)-F | CTCAACTTGGGCCTCTTCTC |
| Kdm6b(Jmjd3)-R | GCCTGTCAGATCCCAGTTCT |
| Kdm6c(Uty)-F | ATTCTGAAGCAATATCAGAC |
| Kdm6c(Uty)-R | TACAGTAATGAGCTGGTTCA |
| Jmjd1c-F | TTTGTTGAAGCTATTGACTG |
| Jmjd1c-R | CACTTTAACAAAAGCAAGCC |
| Kdm4(Jmjd4)-F | GGCCCTTCCAGAAATAAAGACC |
| Kdm4(Jmjd4)-R | CAGGCAGTGGCCATGAACAG |
| Kdm8(Jmjd5)-F | GTGGGGAGAGCCCAGAAGGACATT |
| Kdm8(Jmjd5)-R | GACCCACCCGTTTCCCAAAA |
| Hspap1-F | ACGTAGAAGCTACACTCGAAG |
| Hspap1-R | TAAGCCCAGAACTTGGAATG |
| Kdm7c(Phf2)-F | TCGGCACTTCTCTGTTCTCCC |
| Kdm7c(Phf2)-R | AAATCCAGCCCCTCCGTGTC |
| Kdm7b(Pth8)-F | CTTTCTCTACCTTGGGGACC |
| Kdm7b(Pth8)-R | TAGGGACTCACGTGCTAGGG |
| Fih1-F | TTACTTAACCTCTCTGAGCC |
| Fih1-R | CCAACAACCCTGAGGTAGAT |
| Hairless-F | AGACCTCTGCCCTCTCTGCT |
| Hairless-R | GCTTGGAACACAGCCCAGTC |
| Pla2g4b-F | GCCCAGGCCACACATAATTT |
| Pla2g4b-R | AGTGGTAGCTTTCCATGTGG |
| Loc339123-F | AGCAAGCGACACACACTCAC |
| Loc339123-R | GCACTCAACTCTTCACAGGA |
| Kdm7a(Kiaa1718)-F | ATCTGTGAACTTTGGAGAGG |
| Kdm7a(Kiaa1718)-R | TAGGGACTCACGTGCTAGGG |
| Jarid2-F | AAGTGCTGCTTACATCACTG |
| Jarid2-R | AGTGGATCATAGGACGTTCC |
| Ptdsr-F | AGGTGGATCACTTGAGGTCA |
| Ptdsr-R | CACCACACCTGGCTAATTTT |
|  |  |
| **Genes for cartilage development** |  |
| Col2a1-F | CCAGGGCTCCAATGATGTAG |
| Col2a1-R | GCGGGAGGTCTTCTGTGATC |
| Sox9-F | CCAGCAAGAACAAGCCACAC |
| Sox9-R | TCTCGTTCAGCAGCCTCCAG |
|  |  |
| **Control gene** |  |
| Gaphd-F | TGAAGCAGGCATCTGAGGG |
| Gaphd-R | CGAAGGTGGAAGAGTGGGAG |

**Supplementary Materials and methods.**

**Mouse strains and genotyping**

*Jmjd3*^fl/fl^ mice was described previously(Zhang et al. 2015). *Ezh2*^fl/fl^ mice (Stock No. 000664) (Shen et al. 2008), *Col2a1*-*Cre*^ERT2^ mice (Stock No. 006774)(Nakamura et al. 2006) and *Prx1-Cre* mice (No: 005584)(Logan et al. 2002) were obtained from the the Jackson laboratory. *Utx* ^fl/fl^ mice on C57BL/6 background were previously described(Zheng et al. 2018). All mice were bred and maintained in pathogen-free conditions at the Fourth Military Medical University. Mice of both genders were used for this study. Animal studies were approved by the Institutional Animal Care and Use Committee at the Fourth Military Medical University. Genotyping was tested by PCR on genomic DNA obtained from tails. The primer pairs for genotyping were included in Supplementary Table 1.

**Skeletal preparation**

Whole-mount staining of skeletal preparation from E18.5 embryos was performed as previously described(Zhang et al. 2015). The embryos were skinned, eviscerated, and fixed in 95% ethanol for 4 days. The embryos were stained in alcian blue solution for 24 hours and then washed with 70% ethanol for three times. The embryos were stained in alizarin red solution overnight and transferred into 1% KOH for 1 week. Lastly, the embryos were transferred into 1% KOH/20% glycerol for 2 days and stored in 50% ethanol/50% glycerol.

**Treatment with GSK-J4 or GSK-126**

GSK-J4 (S7070) and GSK-126 (S7061) were purchased from Selleck Chemicals (Houston,TX, USA) and the usage was described previously(Hashizume et al. 2014; McCabe et al. 2012). In brief, GSK-J4 was dissolved in dimethylsulfoxide (DMSO) with a final concentration of 10mg/ml. For the *in vivo* analysis, mice were randomly assigned to vehicle and GSK-J4 treatment groups. One dose of GSKJ4 at 50 mg/kg was administrated by IP. GSK126 or vehicle was administered intraperitoneally at a dose of 100mg/kg in 1:1 v/v GSK126/Captisol mixture resuspended in sterile water with acetic acid to pH4.8. All vehicles were pyrogen-free.

**Tamoxifen treatment**

In preliminary studies, the dose-response of axial skeletal homeotic transformations to tamoxifen (Sigma, T-5648) induced gene recombination was evaluated. Acordding to previous report(Nakamura et al. 2006), three doses of 25mg/kg, 50mg/kg or 100mg/kg of tamoxifen was tested. *Jmjd3^fl/fl^;Col2a1-Cre^ERT2^* mice treated by 50mg/kg instead of 25mg/kg tamoxifen can induced axial skeletal homeotic transformations at a high frequency. Tamoxifen at the dose of 100mg/kg leads most pregnant mice to abortion. Therefore, 50mg/kg tamoxifen was used in the present study if not noted. For determination of embryonic ages, noon on the day of the postcoital plug was taken to be E0.5. Tamoxifen was administered by IP injection as previously described (Nakamura et al. 2006). To bring the drug more rapidly into solution, tamoxifen was dissolved at a concentration of 20 mg/ml in sterile corn oil (Sigma, C-8267) for IP injection. To counteract the estrogen agonist effects of tamoxifen on fetal abortion, progesterone (Sigma, P-3972) dissolved in sterile corn oil was added for IP injections into pregnant females (50mg/kg of progesterone for single injection).

**RNA isolation, reverse transcription, and real-time PCR**

Tissue samples were isolated at the indicated time points, with day E0.5 being noon on the day of the vaginal plug. To screen the regulators of *Hox* gene temporal collear activation in 17 histone methyltransferases and 28 histone demethylases, E7.5, E8.5 and E9.5 embyros were collected respectively for RNA extraction and assays. Among them, E7.5 samples were taken from the whole E7.5 embryos. The E8.5 or E9.5 embryos were decapitated and only the trunks were collected respectively. At least 45 of E7.5 embryos, 25 of E8.5 embryos or 10 of E9.5 embryos in each group were collected respectively. To investigate the effect of GSK-J4 or *Jmjd3* deletion on the mRNA level of *Hox* genes, E8.5 and E9.5 embyros were decapitated and collected respectively. These mouse embryos were treated by vehicle, GSK-J4(50 mg/kg) or tamoxifen (50mg/kg) 12 hours before. Samples were directly lysed in TRIzol (Invitrogen) for RNA isolation following standard protocol. 1 μg RNA was used for reverse transcription by Maxima First Strand cDNA Synthesis kit (Takara). Real-time PCR was performed on ABI Fast7500 with Maxima SYBR Green qPCR Master Mix (Takara). 2;^△△Ct^ method was used for relative quantification. The primer pairs for 17 histone methyltransferases, 28 histone demethylases, *Col2a1, Sox9* and *39 Hox* genes were previously described(Cao et al. 2005; Kondrashov et al. 2011; Ye et al. 2012; Zhang et al. 2015).

**RNA-seq and bioinformatics analyses**

E8.5 embryos were recovered carefully from uteri and isolated from decidua using fine forceps and a needle under a dissecting microscope. The extraembryonic ectoderm was used for genotyping. To investigate the effect of Jmjd3 deletion on the mRNA level of *Hox* genes, we performed RNA sequencing assays with E8.5 *Prx1*-*Cre* (as control) or *Jmjd3 ^fl/fl^*;*Prx1*-*Cre* decapitated embyros. Total RNA was extracted using RNeasy Micro Kit (Qiagen, Cat# 74004) following the manufacturer’s instructions and checked for a RIN number to inspect RNA integrity by an Agilent Bioanalyzer 2100 (Agilent technologies, Santa Clara, CA, US). Qualified total RNA was further purified by RNAClean XP Kit (Beckman Coulter,Inc.Kraemer Boulevard Brea, CA,USA, Cat A63987) and RNase-Free DNase Set (Qiagen, GmBH, Germany Cat#79254). Paired-end libraries were synthesized by using the TruSeq™ RNA Sample Preparation Kit (Illumina, USA) following TruSeq™ RNA Sample Preparation Guide. Briefly, The poly-A containing mRNA molecules were purified using poly-T oligo-attached magnetic beads. Following purification, the mRNA is fragmented into small pieces using divalent cations under 94℃ for 8 min. The cleaved RNA fragments are copied into first strand cDNA using reverse transcriptase and random primers. This is followed by second strand cDNA synthesis using DNA Polymerase I and RNase H. These cDNA fragments then go through an end repair process, the addition of a single ‘A’ base, and then ligation of the adapters. The products are then purified and enriched with PCR to create the final cDNA library. Purified libraries were quantified by Qubit® 2.0 Fluorometer (Life Technologies, USA) and validated by Agilent 2100 bioanalyzer (Agilent Technologies, USA) to confirm the insert size and calculate the mole concentration. Cluster was generated by cBot with the library diluted to 10 pM and then were sequenced on the Illumina NovaSeq 6000 (Illumina, USA). The library construction and sequencing was performed at Shanghai Sinomics Corporation and Shanghai Biotechnology Corporation. For bioinformatics analyses, raw sequence reads were initially processed using FastQC (Babraham Institute, Cambridge, UK) for quality control, and then adapter sequences and poor quality reads were removed using Seqtk. Paired-end sequence files (fastq) were mapped to the mouse genome (mm10) using Hisat2 (Hierarchical Indexing for Spliced Alignment of Transcripts, version 2.0.5). The output SAM (sequencing alignment/map) files were converted to BAM (binary alignment/map) files and sorted using SAMtools (version 1.3.1). Gene abundance was expressed as fragments per kilobase of exon per million reads mapped (FPKM). Stringtie software was used to count the fragment within each gene, and TMM algorithm was used for normalization. Differential expression analysis for mRNA was performed using R package edgeR. R-3.4.3 software was used to draw peak map of *Hox* Transcript profiles and karyoplote R software was used to draw *Hox* genome structure.

**ChIP-qPCR assays**

E8.5 or E9.5 embryos for chromatin immunoprecipitation (ChIP) experiments were decapitated and collected after GSK-J4 (50 mg/kg) or Tamoxifen (50mg/kg) treatment 12 hours before. At least 120 of E8.5 embryos or 40 of E9.5 embryos in each group were collected. Embryonic tissues were collected into 1.5ml EP tube and cut into pieces at 4℃, then digested by 2.5% trypsin into a single cell suspension, and filtered into a new 1.5ml EP tube. The detail procedure ChIP experiments was described previously(Dhar et al. 2016; Zhang et al. 2015). Briefly, cells was cross-linked with 1% formaldehyde for 15 min at room temperature. Glycine with a final concentration of 125 mM was used to deactivate formaldehyde. Cells were lysed using 800μl lysis buffer consisted of 50mM Tris–HCl pH 8.0, 10mM EDTA, 1% SDS, added complete proteinase inhibitors (Roche) and sonicated into small chromatin fragments with an average size of 400 bp. 3 mg of rabbit anti-H3K27me3 (Millipore, #07-449) or anti-Jmjd3 (Abcam, ab85392) antibodies were incubated with sonicated chromatin overnight at 4℃. Subsequently 40 μl protein A/G plus agarose (Santa Cruz Biotechnology) in each EP tube was added and incubated for 2h at 4℃ in total volume of 200 μl RIPA buffer containing 10mM Tris–HCl pH 8.0, 140mM NaCl, 1mM EDTA, 1% Triton X-100, 0.1% SDS and complete protease inhibitor cocktail (Roche). After 5× washing with RIPA buffer and 1× with TE buffer, chromatin was eluted, followed by reverse cross-linking at 65℃ overnight. The DNA fragments were then purified using phenol-chloroform extraction and ethanol precipitation. The purified DNA was quantified by Real-time PCR with Maxima SYBR Green qPCR Master Mix (Takara). 2;^△△Ct^ method was used for relative quantification. The primer pairs for *Hox* gene were previously described(Dhar et al. 2016; Li et al. 2013; Srivastava et al. 2015).

**Western blot analysis**

E9.5 embryos for western blot analysis were decapitated and collected after Tamoxifen (50mg/kg) treatment 12 hours before. The embryonic tissues were sheared, grinded, and lysed into Radio Immunoprecipitation Assay (RIPA) buffer containing protease inhibitor cocktails (Roche, Switzerland). The embryonic tissues were separated by 10%SDS-PAGE gel and transferred onto a PVDF membrane (Millipore). PVDF membranes were blocked with 5% dry milk in Tris-buffered saline for 1h at room temperature. Then the membranes were probed with antibodies against Jmjd3 (Abcam, ab85392,1/500) or beta-tubulin (Sigma Aldrich Inc., 1/3000) antibodies overnight at 4°C. Secondary detection was performed using anti-Rabbit (Millipore, 1/10,000) or anti-Mouse (Millipore, 1/10,000) antibodies at room temperature for 1 h. After being extensively washed with PBS, the protein signals of interest were detected by enhanced chemiluminescence (ECL) and exposure to X-ray film.

**Whole-mount in situ hybridization**

E9.5 embryos were prepared and stained using digoxigenin-11-UTP-labed single-stranded *Jmjd3* or *Ezh2* RNA probes with a DIG RNA labeling kit (Roche) according to the manufacturer’s instructions. Briefly, embryos were hybridized at 65°C overnight, washed, and incubated with 1:1000 dilution of alkaline phosphatase-conjugated antibody (Sigma) at 4°C overnight. The color reaction was performed with nitro blue tetrazolium/5-bromo-4-chloro-3-indolyl-phosphate (Sigma). Images were taken using a stereo microscope (SZX16; Olympus) equipped with a digital camera (DP71; Olympus).

**Data Availability.**

The authors declare that data supporting the findings of this study are available within the article and its Supplementary Information files or from the corresponding author on request. The RNA sequencing data cited in Supplementary Fig. S1,S2 have been deposited in the NCBI SRA database under the accession code PRJNA673586.

**Statistical analysis.**

Statistical analyses were performed using Prism 6 software. Student’s t tests were used and error bars reflect standard deviation (SD) unless otherwise indicated. For multiple comparison analysis, Tukey’s HSD was performed by SPSS 20.0 software. Throughout the study, p < 0.05 was defined as the threshold for significance

**References**

Cao, R., Y. Tsukada, and Y. Zhang. 2005. Role of Bmi-1 and Ring1A in H2A ubiquitylation and Hox gene silencing. Mol Cell 20(6):845-854.

Dhar, S.S., S.H. Lee, K. Chen, G. Zhu, W. Oh, K. Allton, O. Gafni, Y.Z. Kim, A.S. Tomoiga, M.C. Barton, J.H. Hanna, Z. Wang, W. Li, and M.G. Lee. 2016. An essential role for UTX in resolution and activation of bivalent promoters. Nucleic Acids Res 44(8):3659-3674.

Hashizume, R., N. Andor, Y. Ihara, R. Lerner, H. Gan, X. Chen, D. Fang, X. Huang, M.W. Tom, V. Ngo, D. Solomon, S. Mueller, P.L. Paris, Z. Zhang, C. Petritsch, N. Gupta, T.A. Waldman, and C.D. James. 2014. Pharmacologic inhibition of histone demethylation as a therapy for pediatric brainstem glioma. Nature medicine 20(12):1394-1396.

Kondrashov, N., A. Pusic, C.R. Stumpf, K. Shimizu, A.C. Hsieh, J. Ishijima, T. Shiroishi, and M. Barna. 2011. Ribosome-mediated specificity in Hox mRNA translation and vertebrate tissue patterning. Cell 145(3):383-397.

Li, L., B. Liu, O.L. Wapinski, M.C. Tsai, K. Qu, J. Zhang, J.C. Carlson, M. Lin, F. Fang, R.A. Gupta, J.A. Helms, and H.Y. Chang. 2013. Targeted disruption of Hotair leads to homeotic transformation and gene derepression. Cell Rep 5(1):3-12.

Logan, M., J.F. Martin, A. Nagy, C. Lobe, E.N. Olson, and C.J. Tabin. 2002. Expression of Cre Recombinase in the developing mouse limb bud driven by a Prxl enhancer. Genesis 33(2):77-80.

McCabe, M.T., H.M. Ott, G. Ganji, S. Korenchuk, C. Thompson, G.S. Van Aller, Y. Liu, A.P. Graves, A. Della Pietra, 3rd, E. Diaz, L.V. LaFrance, M. Mellinger, C. Duquenne, X. Tian, R.G. Kruger, C.F. McHugh, M. Brandt, W.H. Miller, D. Dhanak, S.K. Verma, P.J. Tummino, and C.L. Creasy. 2012. EZH2 inhibition as a therapeutic strategy for lymphoma with EZH2-activating mutations. Nature 492(7427):108-112.

Nakamura, E., M.T. Nguyen, and S. Mackem. 2006. Kinetics of tamoxifen-regulated Cre activity in mice using a cartilage-specific CreER(T) to assay temporal activity windows along the proximodistal limb skeleton. Dev Dyn 235(9):2603-2612.

Shen, X., Y. Liu, Y.J. Hsu, Y. Fujiwara, J. Kim, X. Mao, G.C. Yuan, and S.H. Orkin. 2008. EZH1 mediates methylation on histone H3 lysine 27 and complements EZH2 in maintaining stem cell identity and executing pluripotency. Mol Cell 32(4):491-502.

Srivastava, S., J. Dhawan, and R.K. Mishra. 2015. Epigenetic mechanisms and boundaries in the regulation of mammalian Hox clusters. Mech Dev 138 Pt 2:160-169.

Ye, L., Z. Fan, B. Yu, J. Chang, K. Al Hezaimi, X. Zhou, N.H. Park, and C.Y. Wang. 2012. Histone demethylases KDM4B and KDM6B promotes osteogenic differentiation of human MSCs. Cell Stem Cell 11(1):50-61.

Zhang, F., L. Xu, L. Xu, Q. Xu, D. Li, Y. Yang, G. Karsenty, and C.D. Chen. 2015. JMJD3 promotes chondrocyte proliferation and hypertrophy during endochondral bone formation in mice. J Mol Cell Biol 7(1):23-34.

Zheng, L., L. Xu, Q. Xu, L. Yu, D. Zhao, P. Chen, W. Wang, Y. Wang, G. Han, and C.D. Chen. 2018. Utx loss causes myeloid transformation. Leukemia 32(6):1458-1465.
